# Calm in the midst of the storm: inferring gene expression stability in flowering plants

**DOI:** 10.3389/fpls.2026.1846139

**Published:** 2026-08-17

**Authors:** Anna V. Klepikova, Alexandra M. Kasianova, Artem S. Kasianov, Aleksey A. Penin, Maria D. Logacheva

**Affiliations:** 1 Center of Bio- and Medical Technology, Moscow, Russia; 2 Laboratory of Plant Genomics, Vavilov Institute of General Genetics, Russian Academy of Sciences, Moscow, Russia; 3 CIBIO, Centro de Investigação em Biodiversidade e Recursos Genéticos, InBIO Laboratório Associado, Campus de Vairão, Universidade do Porto, Vairão, Portugal; 4 Biopolis Program in Genomics, Biodiversity and Land Planning, CIBIO, Campus de Vairão, Vairão, Portugal

**Keywords:** angiosperms, flowering plants, housekeeping genes, multiomics, orthology, stably expressed genes, transcriptomics, ubiquitous genes

## Abstract

**Introduction:**

Gene expression in plants is inherently dynamic, shifting rapidly during development and in response to environmental stimuli. However, many essential cellular processes require remarkable transcriptomic stability. The identity, genomic features, and evolutionary conservation of stably expressed genes (SEGs) in plants across broad evolutionary scales remain poorly understood.

**Results:**

We analyzed comprehensive transcriptome atlases from five species representing major clades of angiosperms (*Arabidopsis thaliana*, *Fagopyrum esculentum*, *Solanum lycopersicum*, *Phalaenopsis equestris*, and *Zea mays*) to identify genes with consistently low expression variation across organs, developmental stages, and environmental conditions. We identified 672 orthogroups that are universally stable across angiosperms; these are predominantly single-copy genes enriched for fundamental cellular functions including DNA repair, mRNA splicing, and translation. Validation using independent datasets and additional species confirmed that stability is maintained across diverse genotypes, conditions, and species. SEGs tend to be longer, have more exons, and exhibit lower GC content than other expressed genes. Using multiomics information available for *Arabidopsis*, we also explored epigenetic and functional characteristics associated with stability. Loss-of-function mutations in SEGs result in lethality far more frequently than in ubiquitously expressed genes, and protein abundance is similarly stable. SEGs have a significantly higher level of gene body methylation and H3K4me1 marks.

**Conclusions:**

Our results reveal several genomic and epigenetic features that distinguish SEGs from ubiquitously expressed genes. They also suggest that SEG stability is not governed by specific regulatory motifs, but is rather “by default” state driven by a distinctive chromatin architecture.

## 1 Introduction

Changes in gene expression are the primary drivers of biological processes in all living organisms. Drastic transcriptomic shifts are essential for development and for responses to external and internal stimuli. At the evolutionary scale, the changes in gene expression level and pattern are associated with the emergence of morphological and physiological innovations (Álvarez-Buylla et al., 2010; Mika et al., 2021; Martín-Zamora et al., 2023). At the same time, many cellular processes (such as DNA repair, protein synthesis, and basic metabolism) are universal across a range of developmental stages and conditions and require remarkable stability of the transcriptome. However, the structure and regulation of the fraction of the transcriptome that mediates this stability is not comprehensively explored. Stably expressed genes (SEGs) (or “housekeeping genes”—these terms are often used interchangeably, though strictly, housekeeping genes represent a broader class of genes, where SEGs are a subset) have been characterized the most extensively in animal model species (especially in human and mouse; Hounkpe et al., 2021). In animals, SEGs are typically associated with specific genomic signatures, including higher gene body methylation (Sarda et al., 2012), lower conservation of promoter sequences (Farré et al., 2007), and shorter gene lengths and fewer introns, which may minimize the metabolic cost and noise of transcription or reflect simpler protein structure (Eisenberg and Levanon, 2003; Vinogradov, 2004; Nie et al., 2010).

In plants, there are several studies focused on within-species gene stability, mostly motivated by practical applications, such as identifying reliable reference genes for qPCR normalization (Czechowski et al., 2005; Yim et al., 2015; Zhuo et al., 2016). In an evolutionary context, there are few studies aimed at understanding the promoter architecture of stable/housekeeping genes versus tissue-specific genes (Das and Bansal, 2019; Yang et al., 2023). Also, a link between expression stability and gene body methylation found in animals (see above) persists in plants as well (Zastąpiło et al., 2026) and presumably has a causative relationship. However, several fundamental research questions persist. First, it remains unclear whether there is a truly universal set of SEGs shared across the deeply divergent lineages of angiosperms. Second, while animal SEGs often exhibit specific structural features, it is unknown if plants (given their unique genome architecture dominated by frequent whole-genome duplications) rely on the same genomic or epigenomic hallmarks to maintain expression stability. Finally, a significant challenge is the distinction between “ubiquitously expressed” genes (present in all tissues) and truly “stably expressed” genes (maintaining constant transcript levels). To address these questions, it is necessary to analyze high-resolution transcriptome maps. Low-resolution or tissue-limited datasets often conflate ubiquity with stability, failing to capture the subtle variations in expression that occur during stress or rare developmental transitions. By using comprehensive RNA-seq datasets that provide full coverage of organs, developmental stages, and environmental conditions, we can isolate the most robustly stable fraction of the genome.

In this study, we utilize high-resolution data from five representative flowering plant species (*Arabidopsis thaliana*, *Fagopyrum esculentum*, *Solanum lycopersicum*, *Phalaenopsis equestris*, and *Zea mays*) to: 1) identify the fraction of genes that maintain stability across a vast range of conditions within a species, 2) assess the evolutionary conservation of these SEGs across the angiosperm phylogeny, and 3) characterize the genomic and epigenomic features associated with expression stability. Flowering plants serve as an ideal model for this inquiry due to their deep morphological divergence and the vast amount of available transcriptomic data covering many distinct lineages (Klepikova and Penin, 2019).

## 2 Materials and methods

### 2.1 Orthogroup inference

Protein sequences of 109 angiosperm species were downloaded from Phytozome 14 (Goodstein et al., 2012) (for the full list of species and names of downloaded files, see **Supplementary File 1**). For the five “core” species, proteins derived from the longest isoform of each protein-coding gene were obtained using the following genome assemblies and annotations: *A. thaliana*—GCA_978657495.1_TAIR12 (Reiser et al., 2026), *F. esculentum*—GCA_965199375.1 (Gunko et al., 2025), *S. lycopersicum*—Slycopersicum_796_ITAG5.0.gene (Phytozome 14), *Z. mays*—Zm-B73-REFERENCE-NAM-5.0 (Hufford et al., 2021), and *P. equestris*—PLAZA database v.4.5 (Cai et al., 2015). OrthoFinder v3.1.4 (Emms and Kelly, 2019) with default settings was used to construct gene orthogroups.

### 2.2 RNA-seq read mapping and counting

Transcriptomic data were analyzed for the five “core” species: *A. thaliana*, *F. esculentum*, *S. lycopersicum*, *Z. mays*, and *P. equestris*. The full list of NCBI SRR IDs of transcriptome samples is presented in **Supplementary Table 1**. Reads were trimmed using Trimmomatic v0.36 (Bolger et al., 2014) with the following parameters “*ILLUMINACLIP:adapters.all:2:30:10 LEADING:20 TRAILING:20 SLIDINGWINDOW:4:15 MINLEN:30*” and mapped on the respective genomes (listed above) with STAR v2.7.10b (Dobin et al., 2013). Only uniquely mapped reads were considered. The read number per gene was counted together with mapping by the “*--quantMode GeneCounts*” STAR option.

### 2.3 Definition of ubiquitously expressed genes

Read counts were normalized on library size using the DESeq median method (Anders and Huber, 2010) for each species separately. A gene was counted as expressed in a sample if it had 16 or more normalized read counts in each biological replicate of the sample (a “strict” threshold; Su et al., 2014). Genes expressed in every sample of a species were considered ubiquitously expressed.

### 2.4 Measurement of expression stability

For each ubiquitously expressed gene, the coefficient of variation (CV) was calculated as the mean expression divided by the standard deviation of expression across all samples of a species.

### 2.5 Gene Ontology enrichment

Gene Ontology enrichment of stably expressed *A. thaliana* genes was calculated using the Database for Annotation, Visualization, and Integrated Discovery (DAVID) tool (Huang et al., 2009; Sherman et al., 2022). Genes expressed in all samples of *A. thaliana* (i.e., ubiquitous genes) were used as a background for the analysis. Terms with fold enrichment ≥2 and FDR <0.05 were considered overrepresented in stable genes compared with ubiquitous ones.

### 2.6 Proteome analysis

A proteome map of *A. thaliana* was used to assess stability of SEGs at the proteome level (Mergner et al., 2020). Intensity-based absolute quantification (iBAQ) values were extracted for stable and ubiquitous genes from supplementary materials 41586_2020_2094_MOESM4_ESM.xlsx. The CV of iBAQ values was calculated on a proteome map as described above for transcriptome maps.

### 2.7 Codon usage analysis

DAMBE7 software (Xia, 2018) was used to calculate the effective number of codons for stable and ubiquitous genes of each species. CDS sequences for the studied genes were used as input for the software (in case of multiple isoforms, the longest one was taken in the analysis). The expected effective number of codons was calculated using DAMBE7 as well. The R package “coRdon” (Bioconductor, 2026) was used to calculate codon frequencies for stable and ubiquitous genes of each species. Then, to generate PR2 bias plots, the frequencies of each nucleotide at the third position of the codon were calculated as the sum of respective codon frequencies. The frequency of the A nucleotide was divided by the sum of A and T nucleotide frequencies, and the frequency of the G nucleotide was divided by the sum of G and C nucleotide frequencies, resulting in A3/(A3+T3) and G3/(G3+C3) values in the PR2 bias plot. For neutrality plots, frequencies of GC content were calculated at each position of the codon, naming GC1, GC2, and GC3, respectively. GC12 was calculated as the mean of GC1 and GC2. Relative synonymous codon usage (RSCU) was calculated as follows: for each group of synonymous codons, codon frequencies were summed and then divided by the number of synonymous codons in the group, resulting in uniform codon frequencies. RSCU was calculated as the ratio of observed codon frequencies to expected uniform codon frequency.

### 2.8 Cytosine methylation analysis

To explore cytosine methylation, several datasets were used: 20 ecotypes were randomly selected from 1001 Epigenomes data on *Arabidopsis* (Kawakatsu et al., 2016), 24 samples of developing and germinating seeds (Kawakatsu et al., 2017), 6 samples of ovules and anthers (Wu et al., 2025), 12 samples treated with salicylic acid together with controls (Margaritopoulou et al., 2026), 12 samples subjected to nutritional stress, and 4 samples treated with heat condition; 91 samples were analyzed in total. For the full list of samples, see **Supplementary Table 2**. Reads were trimmed with Trim Galore v2.2.0 with default settings (Babraham Bioinformatics, 2026). Read mapping, read deduplication, and methylation calling were performed with Bismark v0.25.1 (Krueger and Andrews, 2011). Only fully methylated positions (with >90% of reads supporting methylated C) were considered for the subsequent analysis. For each sample and stable and ubiquitous gene, the number of methylated C in three contexts (CG, CHH, and CHG) was calculated together with the total number of C in these three contexts in 5′-UTR, CDS, introns, and 3′-UTR. For each context, the ratio of methylated C was calculated as the number of methylated C to the total number of C in the context.

### 2.9 Histone modifications analysis

BigWig files describing the level of histone modifications at certain chromosome positions were downloaded from the Plant Chromatin State Database (Liu et al., 2018). BigWig files were converted to bedGraph using the bigWigToBedGraph tool (Anaconda, 2026). The levels of histone modifications were counted and averaged for the 5′-UTR, gene body, and 3′-UTR of each protein-coding ubiquitous or stable gene.

### 2.10 ATAC-seq analysis

BED files with ATAC-seq peak calling results were downloaded from the PlantCADB database (Ding et al., 2023) (for the list of samples used, see **Supplementary Table 3**). For each gene, the number of ATAC-seq peaks located in the 1,000-bp region upstream of the TSS was calculated in each sample. If a gene had at least one ATAC-seq peak in both replicates of any sample, the gene is considered to have a promoter region with open chromatin.

### 2.11 Overrepresentation of cis-regulatory motifs in gene promoters

Promoter regions, i.e., 1,000 bp sequence upstream of the annotated start of the longest protein-coding isoform of stable and ubiquitous genes, were extracted. Promoter coordinates were generated using BEDTools flank v2.31.1 with the following options “*-l 1000 -r 0 -s*” (Quinlan and Hall, 2010). The sequences were extracted from the reference genomes using the BEDTools getfasta with parameters “*-s -name*.” Overrepresentation of transcription factor binding motifs was identified using motif models from the CIS-BP database (Weirauch et al., 2014). Motif enrichment analysis was performed using the findMotifs.pl module of HOMER v5.1 with the parameters “*-len 8,10,12 -S 10 -basic*” (Heinz et al., 2010). Analyses were performed separately for each of the “core” species and on a dataset combining promoter sequences from all five species.

### 2.12 Data visualization

The R package “ggplot2” (Wickham, 2016) was used to create histograms, violin plots, pie charts, box plots, scatter plots, and distribution plots. Expression level was normalized on GeTMM to remove the influence of both library size and gene length (Smid et al., 2018). For heatmap visualization, the R package “complexHeatmap” was used (Gu et al., 2016). Venn diagram was drawn with the UGent Bioinformatics & Evolutionary Genomics Venn tool (Bioinformatics & Evolutionary Genomics, 2026). The R package “chromoMap” (Anand and Rodriguez Lopez, 2022) was used to analyze the distribution of stable genes across *A. thaliana* chromosomes. Two input files were used: the first one with coordinates of chromosomes (i.e., chromosome length) and chromosome centromeres, and the second file with gene coordinates.

## 3 Results

### 3.1 The inference of stably expressed orthogroups

We selected 114 angiosperm species with high-quality genome assemblies and annotations available; these species represent main evolutionary lineages within flowering plants. At the first step, we inferred gene orthogroups using OrthoFinder (Emms and Kelly, 2019) and excluded single-gene and single-species orthogroups from the analysis. The 45,900 remaining orthogroups comprised 3,964,165 genes with 2–10,647 genes and 2–114 species per orthogroup (for the description of orthogroups, see **Supplementary File 1**).

Out of the species used for orthology inference, we selected five species: *F. esculentum* (buckwheat, caryophyllids, eudicots), *A. thaliana* (thale cress, rosids, eudicots), *S. lycopersicum* (tomato, asterids, eudicots), *Z. mays* (maize, grass monocots), and *P. equestris* (horse phalaenopsis, Orchidaceae, non-grass monocots) (Klepikova et al., 2016, Klepikova et al., 2021; Stelpflug et al., 2016; Penin et al., 2019, Penin et al., 2021) for which highly detailed transcriptome maps are available (for the list of samples, see **Supplementary Table 1**; for the description of expression dataset quality, see **Supplementary File 2**). A total of 13,876 out of 45,900 orthogroups included at least two genes of two or more aforementioned species (these orthogroups covered 86.7% of all genes in the analysis). Based on the gene expression data for the five well-studied “core” species, we retained for further analysis only those genes that are expressed in all replicates of every sample—ubiquitously expressed genes (see *Materials and methods*). This filtering step retained only ~10% of the orthogroups: 1,539 out of 13,876 orthogroups had all genes of five species expressed in all samples.

**Figure 1:**
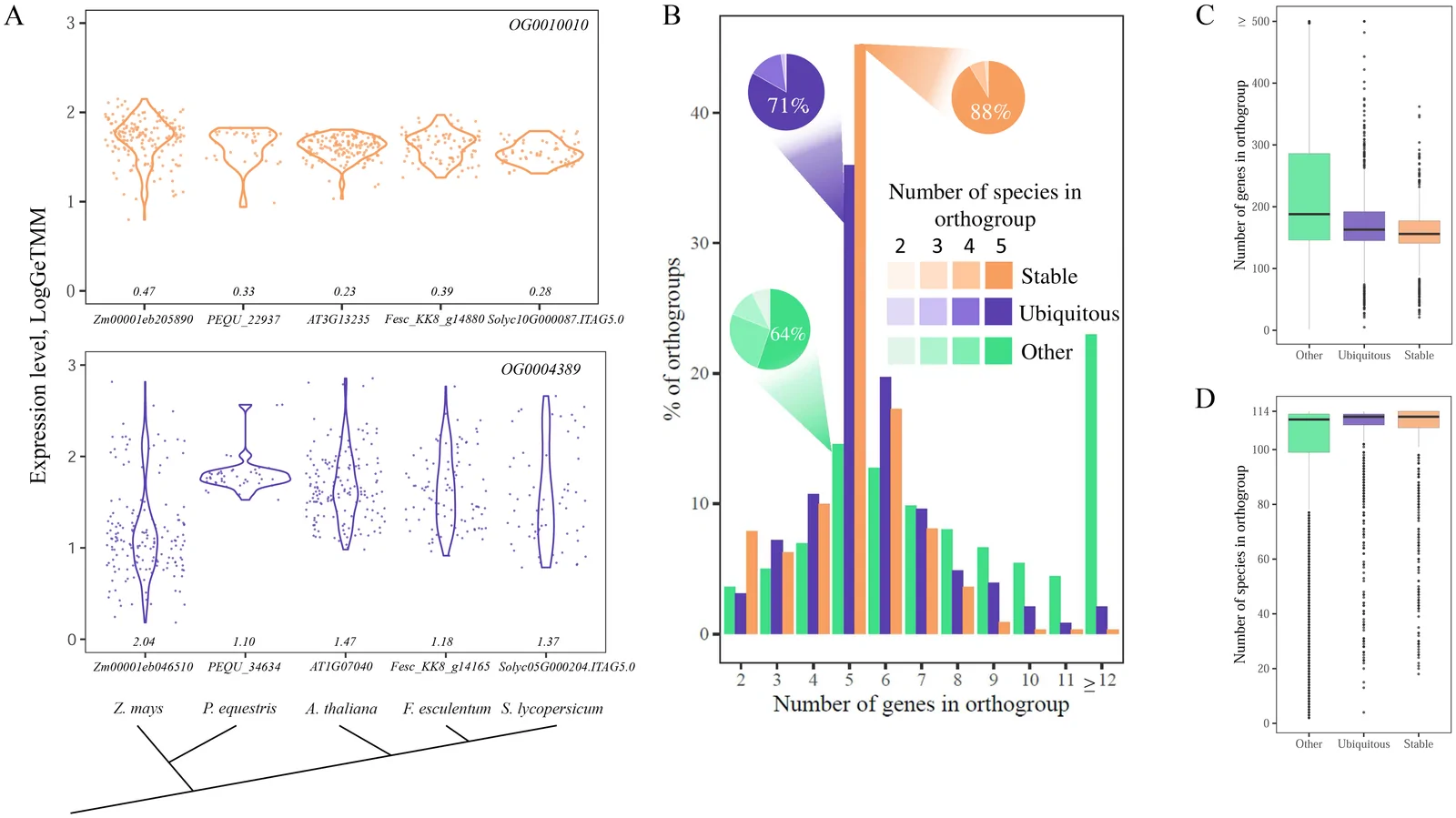
The description of orthogroups. **(A)** The gene expression levels of stable and ubiquitous orthogroups. The upper panel represents the expression level of genes from a randomly selected stable orthogroup; the middle panel represents the expression level of genes from a randomly selected ubiquitously expressed but unstable orthogroup. Numbers above the gene IDs are CV values. Gene expression level is normalized on the library size and gene length (GeTMM values). The lower panel is a schematic view of the phylogeny of the studied species. **(B)** The distribution of orthogroup size considering only genes from five “core” species. Stable orthogroups (orange) are orthogroups with all genes stable; ubiquitous orthogroups (violet) are orthogroups with all genes expressed in all samples (excluding stable orthogroups); and the other orthogroups (green) are all the remaining orthogroups (or orthogroups where some genes are not expressed in all samples). Pie charts represent the ratio of five-gene orthogroups with different numbers of species. The percentage depicted on pie charts describes orthogroups with five genes and five species, i.e., orthopentades. **(C)** The distribution of gene number (orthogroup size) in orthogroups considering genes of all studied species. **(D)** The distribution of species number in orthogroups considering genes of all studied species.

As a measure of gene expression stability, we used CV (Klepikova et al., 2016). To identify SEGs, we applied a relaxed threshold (CV lower than 0.75) due to the large evolutionary distances between species. Under this definition, 672 orthogroups consisted of SEGs (see example of a stable orthogroup in **Figure 1A**). A striking feature of the fraction of genes that are ubiquitously expressed (either stably or not) is that they belong prevalently to the orthogroups that have five genes (considering only five “core” species, **Figure 1B**). Within such orthogroups, the largest fraction is orthopentades—orthogroups with one gene from each of the five species, i.e., the groups of single-copy genes. This trend toward single-copy is more pronounced in stable orthogroups: they have a significantly higher number of orthopentades than ubiquitously expressed or other genes (**Figure 1B**, Fisher’s exact test *p*-value = 2.924e−06 and <2.2e−16, respectively). Stable orthogroups comprised 3,410 genes, which were almost equally distributed between species (from 648 *Phalaenopsis* to 707 *Arabidopsis* genes); the majority of stable orthogroups contained all five species, congruent with the high number of orthopentades.

The overall size of stable and ubiquitous orthogroups was close to the number of analyzed species, i.e., 114 genes (median 156 and 163, respectively, **Figure 1C**), while other orthogroups varied greatly in size (from 2 to 10,647 genes with a median of 188). With respect to species number, stable and ubiquitous orthogroups had almost all species included (72.9% and 72.6% of stable and ubiquitous orthogroups had at least 110 species with a median of 112), and only 61.4% of other orthogroups had 110 species or more (**Figure 1D**). Thus, SEGs tend to be a single copy at the level of all angiosperm plants.

### 3.2 The consistency of stable orthogroups

Since the identification of stable orthogroups relies on a threshold in CV, some orthogroups can fall out of the fraction of stable ones because the coefficient of variation for a certain member of the orthogroup is slightly higher than the threshold. Taking this into account, we examined whether all potentially stable orthogroups were found. All but one of the genes of the “core” species were stable in 455 orthogroups with 187 orthopentades among them (**Supplementary Figure 1A**). The most unstable genes from these orthogroups had high coefficients of variance: the first quartile of the distribution was 0.87/0.84 (for orthopentades/other orthogroups, respectively, **Supplementary Figure 1B**). Thus, there are no “almost-stable” orthogroups. Though the genes that were unstable within these “all-but-one stable” orthogroups had no specific Gene Ontology (GO) enrichment, the majority of unstable genes had a pattern shifted to anthers (in *A. thaliana*, *P. equestris*, *F. esculentum*, *Z. mays*), proliferating tissues (*P. equestris*, *F. esculentum*, *Z. mays*), or senescent organs (*A. thaliana*) (**Supplementary Figures 1C–G**).

In order to provide direct testing of orthogroups identified as stable based on five species analysis, we analyzed gene expression of *Cucumis sativus* (cucumber), a species for which a wide sampling of RNA-seq datasets is available but which was not included in our core set used for the identification of SEGs (**Supplementary File 3**). A total of 672 stable orthogroups included 704 cucumber genes (most of the orthogroups included one cucumber gene, congruent with the tendency of SEGs to be single-copy). A survey of expression stability has shown that 92% of them were stably expressed.

Another important question about SEGs is whether the stability is robust with respect to the sample choice. Transcriptome maps (including the ones used for stability inference in this study) usually represent samples standardized in terms of external conditions and genotypes. In order to explore the robustness of SEGs, we collected five additional sets of publicly available RNA-seq experiments on *A. thaliana* and *Z. mays* (945 and 1,213 samples, respectively). These experiments include leaf samples of different accessions, a detailed atlas of root, biotic and abiotic stress treatments, and supplementation of plants with vitamins (**Supplementary Table 4**). Each of the datasets was merged with a transcriptome map, and coefficients of variance were recalculated. The majority of SEGs retain stability in all datasets for both *A. thaliana* and *Z. mays* (**Table 1**). Although 11% of all *Arabidopsis* SEGs and 16% of maize SEGs became unstable in at least one combination of transcriptome map and public dataset (**Table 2**), 627 A*. thaliana* and 573 *Z. mays* genes were uniformly expressed across a great variety of plant tissues, organs, environmental conditions, and ecological interactions, confirming their status as universal SEGs.

**Table 1:** Number of SEGs retaining stability in the combination of transcriptome map and additional dataset.

Dataset	*A. thaliana* (707 SEGs)	*Z. mays* (685 SEGs)
Leaves of different accessions	702 (99%)	664 (97%)
Detailed root altas	661 (94%)	654 (95%)
Infection of pathogens	702 (95%)	634 (93%)
Abiotic stress treatments	662 (94%)	633 (92%)
Vitamin supplementation	705 (99%)	655 (96%)

**Table 2:** Preservation of expression stability across plant tissues and environmental conditions.

Number of datasets where SEG was stable	A number of genes remained stable in a certain number of dataset combinations	
	*A. thaliana* (707 SEGs)	*Z. mays* (685 SEGs)
5	627 (88.7%)	573 (83.6%)
4	61 (8.6%)	72 (10.5%)
3	16 (2.3%)	17 (2.5%)
2	2 (0.3%)	15 (2.2%)
1	1 (0.1%)	6 (0.9%)
0	0 (0.0%)	2 (0.3%)

### 3.3 SEGs at the proteome and organism levels

**Figure 2:**
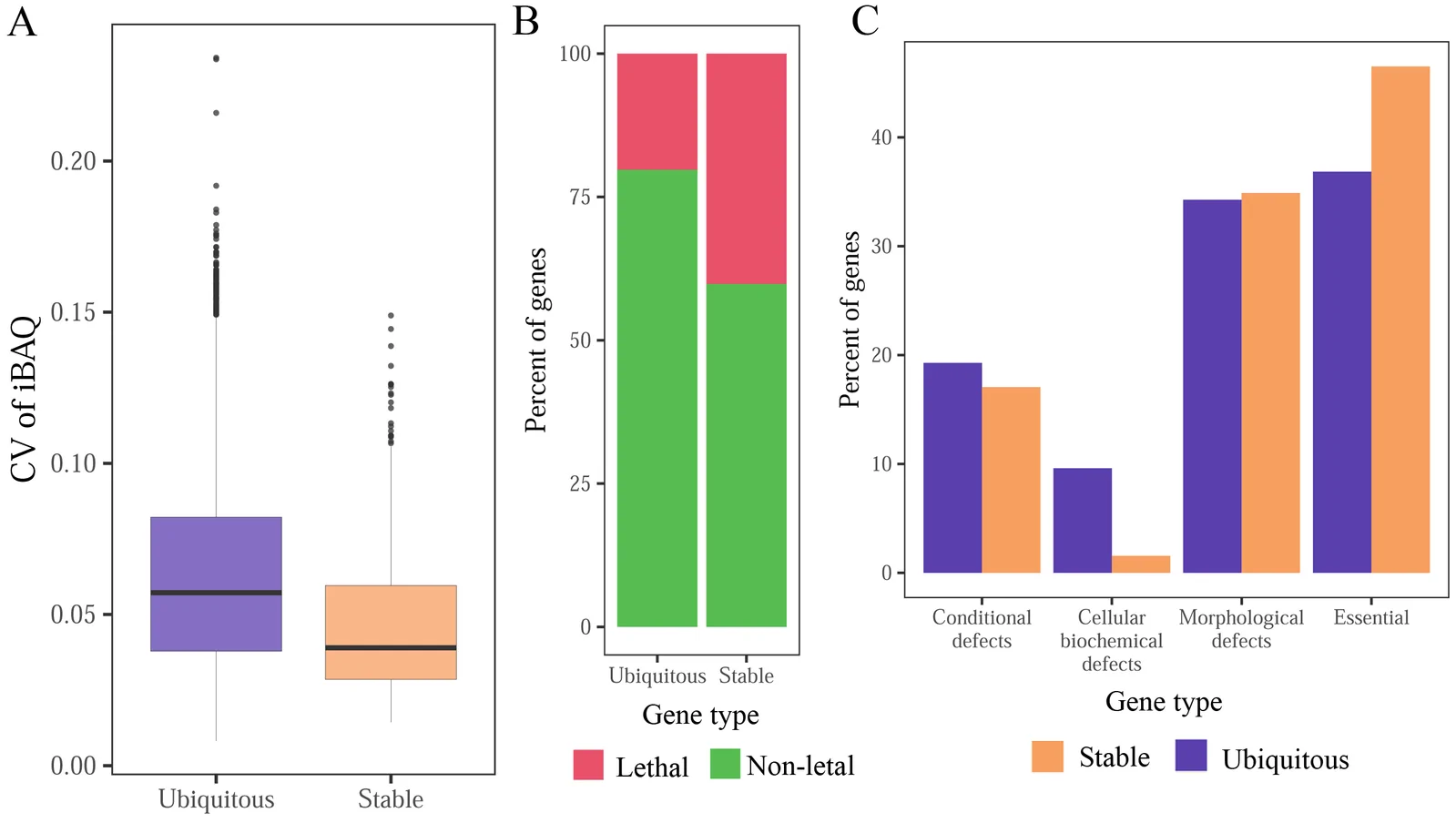
Functional characteristics of genes. **(A)** The distribution of CV of protein level across various *Arabidopsis* organs for stable and ubiquitously expressed genes. **(B)** Percentage of stable and ubiquitous genes whose loss-of-function mutations cause plant lethality. All genes that were not marked as “lethal” were considered non-lethal. **(C)** Percentage of stable and ubiquitously expressed genes classified by the outcome of mutations altering gene function into four categories: essential, with loss of function leading to plant lethality; various morphological defects; disturbance of biochemical processes; and defects dependent on the environment. Asterisks indicate statistically significant differences between distributions of expressed and stable genes using the Mann–Whitney test with *** for *p*-values lower than 1.0e−10, ** 1.0e−10 < FDR < 1.0e−5, and * 1.0e−5 < FDR < 0.05.

The increasing knowledge on post-transcriptional regulation and the analysis of proteomics data show that the expression measured at the level of RNA does not always accurately reflect the abundance of proteins (Greenbaum et al., 2003, reviewed in Liu et al., 2016). Proteomic studies usually lag behind transcriptomic studies; this is the case for plants as well, although the situation begins to change. Recently, a proteome map was published for a model plant of *A.thaliana* (Mergner et al., 2020). This allows us to assess whether SEGs are stable at the proteome level in this species. Indeed, we found that the coefficient of variance was significantly lower for SEGs in comparison with ubiquitous genes (**Figure 2A** Mann–Whitney test *p*-value < 2.2e−16). This shows that the stability of SEGs is maintained not only at the level of transcription but also at the post-transcriptional level.

Such stability implies an essential role in the life of an organism. Indeed, Gene Ontology enrichment analysis for *Arabidopsis* SEGs shows that they are associated with such basic cellular functions as DNA repair, mRNA splicing, translation initiation, and protein transport and degradation (**Supplementary Table 5**). The availability of multiple *Arabidopsis* knockout mutants allows us to directly compare the consequences of loss of function of SEGs and other genes. We analyzed the importance of SEGs using two datasets: the list of *Arabidopsis* genes whose loss-of-function mutations lead to lethality (Lloyd et al., 2015) and the classification of *Arabidopsis* genes by the outcome of loss-of-function mutations (Lloyd and Meinke, 2012). In the latter classification, the genes that are classified as essential (ESN) are almost completely nested into the group of genes with lethal mutations from the former classification (675 out of 719) (**Supplementary Figure 2**).

The loss-of-function mutations in SEGs led to plant lethality far more frequently than in other ubiquitous genes (40% compared to 20%, Fisher’s exact test *p*-value < 2.2e−16, **Figure 2B**). The analysis of the second dataset leads to the same conclusions: mutations in the majority of SEGs resulted in lethality or morphological defects (**Figure 2C**).

### 3.4 Expression and structural characteristics of SEGs

**Figure 3:**
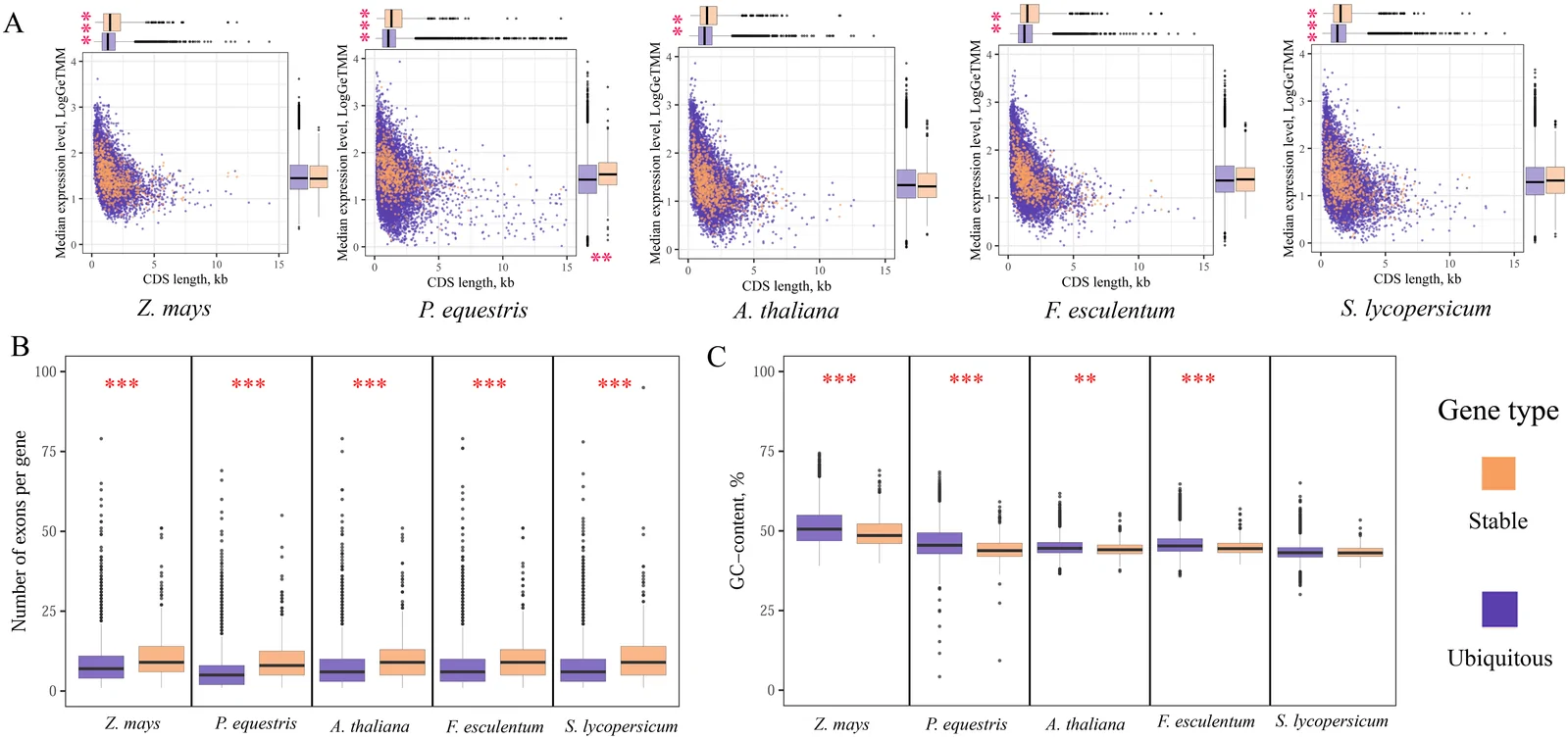
Structural characteristics of genes. **(A)** Scatter plots of ubiquitous and stable genes based on CDS length and median expression level. Each dot represents a gene; expression level was normalized on GeTMM to remove the influence of both library size and gene length. The box plot on the top of each plot represents the distribution of CDS length of a species, and the box plot on the right side of each plot represents the distribution of median expression level of species genes. **(B)** The distribution of gene exon numbers for each species. **(C)** The distribution of gene GC content for each species. Asterisks indicate statistically significant differences between distributions of expressed and stable genes using the Mann–Whitney test with *** for FDR-corrected *p*-values lower than 1.0e−10, **1.0e−10 < FDR < 1.0e−5, and *1.0e−5 < FDR < 0.05.

In order to gain insight into the mechanisms that support the stability of SEGs at the transcriptional and translational levels, we compared several genomic and epigenomic characteristics of angiosperm-scale stable genes (i.e., genes from 672 orthogroups where all genes are stable) and ubiquitous genes (i.e., genes expressed in all samples excluding SEGs). This allowed us to find features associated with stability. First, SEGs of all species were longer than ubiquitous genes in terms of the median length of CDS (**Figure 3A**), exon number (**Figure 3B**), and average intron length (**Supplementary Figure 3**). The GC content of stable genes was lower, with a significantly smaller standard deviation for four species excluding tomato (**Figure 3C**).

The median expression levels of SEGs and ubiquitous genes did not statistically differ for *Z. mays*, *A. thaliana*, *F. esculentum*, and *S. lycopersicum*, while for *P. equestris*, SEGs were expressed at a higher level (**Figure 3A**). Notably, SEGs tend to have more similar expression values: in four species, the standard deviation of median expression level was 2.5–4.9 times lower for SEGs compared with ubiquitous genes (Levene test’s *p*-values were 1.74e,−05 for *Z. mays*, 2.06e−03 for *F. esculentum*, and 1.73e−04 for *A. thaliana*. *P. equestris* and *S. lycopersicum* did not show a significant difference).

Another important feature that is known to be correlated with transcript abundance is codon usage. Synonymous codons can be used unequally in different species or certain genes of a species, a feature called codon usage bias (Hanson and Coller, 2018). We explored codon usage bias in stable and ubiquitous genes using effective number of gene codons (**Supplementary Figure 4A**), PR2 bias plots (**Supplementary Figure 4B**), and neutrality plots (**Supplementary Figure 4C**). We found mild differences in codon characteristics of stable and ubiquitous genes: codon usage of both gene types is influenced by the mutation process rather than natural selection, and GC content in the third position of the codon was lower for SEGs, matching overall GC content. RSCU is the ratio of estimated codon frequency to the expected frequency if all synonymous codons were used equally. RSCU higher than 1.6 is considered overrepresented. The main trend for the four species excluding *Z. mays* was the increasing codon usage bias in SEGs (**Supplementary Table 6**).

### 3.5 Epigenetic profiles of SEGs

**Figure 4:**
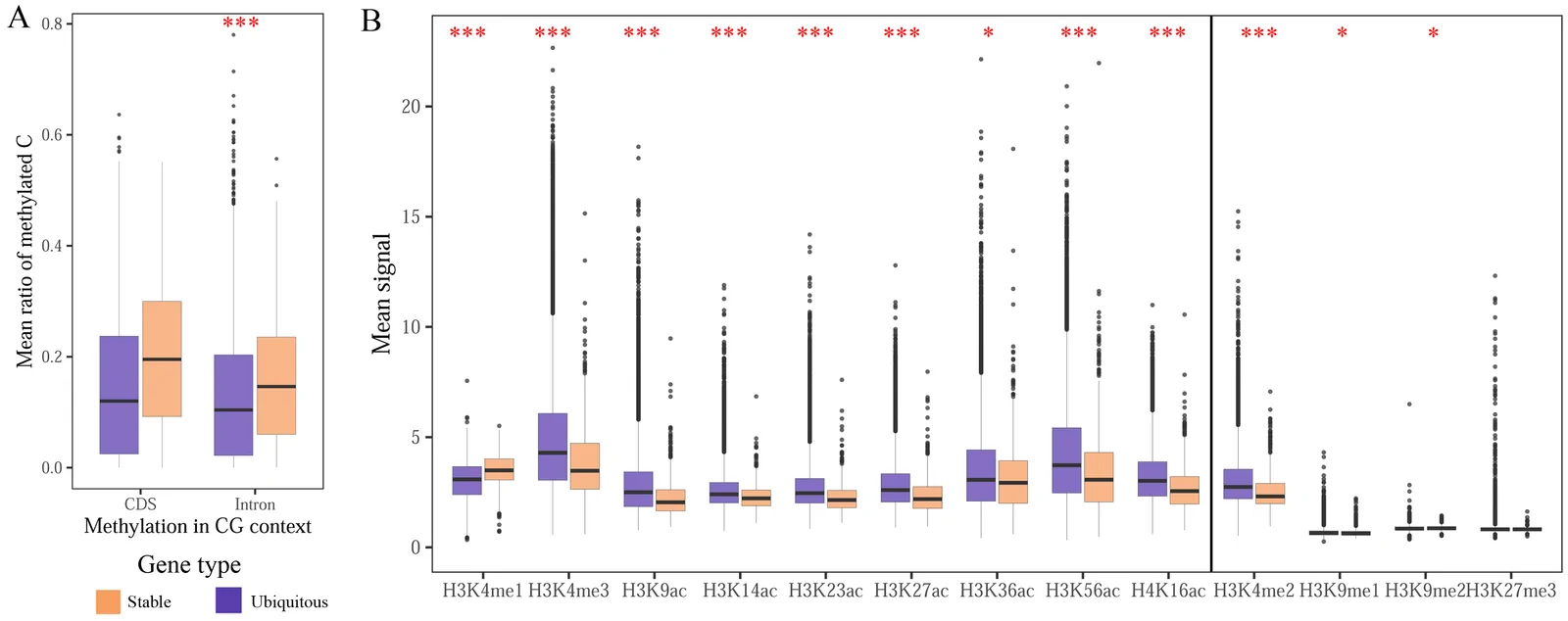
Chromatin characteristics of genes. **(A)** Averaged across 91 samples, the ratio of methylated cytosines to all cytosines in CG contexts of CDS and introns of ubiquitously expressed and stable *A. thaliana* genes. Ratios were averaged across 91 studied samples. **(B)** The distribution of histone modifications in the gene body of ubiquitously expressed and stable *A. thaliana* genes. The vertical solid line separates active histone marks (left part) from repressive ones (right part). Asterisks indicate statistically significant differences between distributions of expressed and stable genes using the Mann–Whitney test with *** for FDR-corrected *p*-values lower than 1.0e−10, **1.0e−10 < FDR < 1.0e−5, and *1.0e−5 < FDR < 0.05. .

Gene expression level and pattern are dependent on the epigenetic state of a gene locus, in particular, on the level of cytosine methylation and histone modifications. In plants, cytosine methylation occurs in three functionally different contexts: CG, CHH, and CHG, where H stands for A, C, or T (Gallego‐Bartolomé, 2020). We used a wide variety of epigenomics data, including ecotypes from 1001 Epigenomes data on *Arabidopsis* (Kawakatsu et al., 2016), various plant organs, and treatments with multiple stress conditions (Kawakatsu et al., 2017; Wu et al., 2025; Margaritopoulou et al., 2026), to explore cytosine methylation in angiosperm-scale stable genes in comparison with genes expressed across whole plants, but not universally stable. While cytosine methylation in UTRs was not detected in the majority of genes (only 8%–41% of genes were methylated in at least one sample depending on context), CDS and introns of SEGs showed significantly higher methylation levels in the CG context (**Figure 4A**). Notably, the variation of cytosine methylation across the studied samples (assessed with CV) was significantly lower for SEGs in the CG context (**Supplementary Figure 5**). The level of CHG and CHH methylation was low both in CDS and introns.

We analyzed different kinds of methylation and acetylation of histones 3 and 4 lysines using data from the Plant Chromatin State Database (Liu et al., 2018). We did not find differences in histone mark distribution in both the 5′- and 3′-UTRs of ubiquitous and stable genes, with one exception (**Supplementary Figures 6A,B**): H3K4me2, the repressive mark in euchromatin, was more frequent in ubiquitous genes compared with stable ones. In contrast, almost all histone modifications in the coding region of genes differed between stable and ubiquitous genes (**Figure 4B**). Methylation of lysine 4 of histone 3 (H3K4me1) was more pronounced in SEGs, while all the other marks associated with active transcription were more frequent among the ubiquitous genes. As expected, all three histone modifications, which mark heterochromatin and repression of transcription, were found at a very low level in both ubiquitous and stable genes.

Supporting the observation on histone marks, the analysis of ATAC-seq data showed the association of SEGs with open chromatin. We used ATAC-seq peaks for roots, seedlings, and leaves deposited in the database PlantCADB (Ding et al., 2023) (for the list of samples used, see **Supplementary Table 3**). For each gene, we calculated the number of ATAC-seq peaks located in the 1,000-bp region upstream of the TSS and considered a gene to have a promoter region with open chromatin if a gene had an ATAC-seq peak in both replicates of any sample. We did not find a difference between SEGs and ubiquitously expressed genes (93.5% of SEGs had an open chromatin in promoter regions compared with 92.1% of ubiquitous genes; among all the other genes, 64.5% were associated with open chromatin). As open chromatin is distributed unequally along the chromosomes, we analyzed the possibility of SEGs being organized in spatial clusters on chromosomes. However, the analysis of gene distribution across chromosomes with the chromoMap R package showed its uniformity (**Supplementary Figure 7**).

Finally, we analyzed the overrepresentation of cis-regulatory motifs recognized by various transcription factors in promoter regions of SEGs compared with ubiquitous genes. Surprisingly, we did not find any motif to be more frequent in SEG promoters than in the promoters of ubiquitous genes, indicating the absence of common regulation of expression stability.

## 4 Discussion

Using a balanced dataset of transcriptome data representing main evolutionary lineages of flowering plants, we found the groups of genes that are stable not only across organs and conditions but also across species. Previous studies on stable genes were mostly focused on within-species stability (Zhuo et al., 2016; Wang et al., 2017; Gu et al., 2023) or paired comparisons between human and model animals (Lin et al., 2019a; Hounkpe et al., 2021). Our study represents a step forward in putting the question of gene expression stability in the evolutionary context and using multiomics data available for a model plant species. First, our analysis highlighted that the stability of expression is strongly associated with single-copy genes present in all species studied. This complements earlier findings on tissue-specific genes: they are, vice versa, associated with duplicated genes—a phenomenon that is presumably common for plants and animals (Ha et al., 2009; Huerta-Cepas et al., 2011; Mantica et al., 2024). There are several factors that can affect conclusions on the stability of gene expression—in particular, the set of species and samples taken into analysis. We thus corroborated our findings using two lines of evidence. First, we added in the analyses a species outside of our five-species core set (*C. sativus*) and found that more than 96% of orthogroups remained stable. Second, for those species for which a vast amount of transcriptomic data not used for initial inference of stable genes is available—*Arabidopsis* and maize—we added several large datasets in order to see how this will affect gene stability. These datasets include biotic and abiotic stresses, different genotypes, and vitamin supplementation. For most datasets, the decrease in the fraction of stable genes was at the level of 1%–4%; overall, more than 86% of SEGs retained stability. It is important to note that the variation of conditions in these datasets included not only the key experimental condition (e.g., a certain stress) but also a number of other aspects of plant growth (temperature of growth, day length, laboratory or open-ground). This emphasizes that the SEGs found in our study are universally stable. The identification of SEGs is essential for the development of many methodologies, e.g., qPCR (Vandesompele et al., 2002; Andersen et al., 2004) and single-cell transcriptome analysis (Lin et al., 2019b; Deeke and Gagnon-Bartsch, 2020). Also, we suggest that these genes can be used as an analog of BUSCO datasets for plant *de novo* transcriptome assemblies. Indeed, a conventional practice for quality control of genome assemblies is the search for core genes—the ones that are thought to be universally present in the genome (this approach was first adopted in Parra et al., 2007), but now replaced by a newer pipeline, BUSCO (Simão et al., 2015; Tegenfeldt et al., 2025). This approach is hardly applicable to transcriptome assemblies, because the set of transcripts is tissue/condition-specific and may not include the core genes. Our inference of stable genes allows us to circumscribe the set of genes that is expected to be present in any transcriptome assembly, thus contributing to the improvement of assembly quality control.

The availability of multiomics data for *Arabidopsis* allowed us not only to identify stable genes but also to explore a number of genomic and functional characteristics associated with stability. This lays a foundation for the mechanistic exploration of the maintenance of expression stability and its significance for the representation of genotype in phenotype. First, at an organismal level, within SEGs, the fraction of essential genes—the ones that result in a lethal phenotype when disrupted—is significantly higher than for other genes expressed in all samples. Earlier studies established an association between essential genes and single-copy genes (Lloyd and Meinke, 2012; Lloyd et al., 2015). The authors also noted that these genes typically exhibit high and broad expression levels. Their definition of expression breadth was based on the number of samples where a gene is expressed; in the same terms, all the genes listed in our study as ubiquitous are broadly expressed. A significant difference between the fraction of essential genes in SEGs and ubiquitous genes emphasizes the uniqueness and essentiality of SEGs at an organismal level. Expression levels and profiles are known to be affected by a number of epigenetic features—e.g., chromatin accessibility, histone modification marks, and cytosine methylation (Miller and Grant, 2013; Wang et al., 2022). In terms of epigenetic profiles, while SEGs closely resemble genes with detectable yet unstable expression, they also exhibit unique features that likely contribute to their stability. First, stable genes have a significantly higher level of gene body methylation. The association of gene body methylation with actively expressed genes is known for both plants and animals (Sarda et al., 2012; Zilberman, 2017). A recent large-scale study on natural populations of *Arabidopsis* has revealed that gene body methylation definitely contributes to interpopulation expression stability (Shahzad et al., 2025). Our findings are congruent with this. Regarding histone modification, there are also a number of significant differences between SEGs and ubiquitous genes. Most of those differences concern gene body regions. In particular, there is an increase in H3K4me1 levels in SEGs. Other histone modifications either do not show significant differences or have decreased levels. H3K4me1 is a well-known activating mark; it is strongly associated with actively transcribed genes (Zhang et al., 2009). While generally all variants of H3K4 methylation (me1, me2, and me3) are regarded as marks of transcriptionally active chromatin (Le et al., 2025), there are subtle differences in their localization, function, and interaction with other epigenetic mechanisms. In particular, H3K4me2- and H3K4me3-containing regions are depleted in GC gene body methylation, while in contrast, it is highly enriched in H3K4me1-containing regions (Zhang et al., 2009). Congruent with that, we observe a higher level of GBM in SEG and a higher level of H3K4me1, but not other types of H3K4 methylation. For H3K4me2, the situation is even more complex—there is a report on rice showing its function as a repressive mark (Liu et al., 2019). Also, in *Arabidopsis*, there is accumulating evidence that it is in fact repressive (Mori et al., 2023; Noyori et al., 2025). Congruent with this, the level of H3K4me2 in SEG is significantly lower.

At the same time, chromatin accessibility does not differ between stable and expressed genes. When comparing broadly expressed versus tissue- or stage-specific genes, there is a significant difference (Boyle et al., 2008; Delandre et al., 2022). Indeed, chromatin accessibility determines the possibility of being expressed, but the actual levels of expression are fine-tuned by other mechanisms.

The absence of significantly enriched cis-regulatory motifs in the promoters of SEGs was an unexpected finding. It suggests that despite being conserved at the level of flowering plants, expression stability is not governed by universal regulatory elements. This lack of motif enrichment distinguishes SEGs from tissue-specific or stress-responsive genes, which typically rely on the presence of specialized elements to coordinate dynamic transcriptomic shifts, and many of these elements are evolutionarily conserved (Spensley et al., 2009; Gasch et al., 2016). Instead, we propose that the stability of these core genes may be a fundamental property of their constitutively accessible chromatin and the notable absence of repressive marks like H3K4me2, which ensures continuous recognition by the general transcriptional machinery. These results lead us to the hypothesis that the stability of plant SEGs might be maintained through a decentralized regulatory logic, where constitutive activity is a default state supported by genomic architecture rather than specific promoter motifs. However, currently available chromatin accessibility datasets for plants are quite scarce; further genome-wide analysis and functional studies are necessary to support this hypothesis and to provide a mechanistic explanation for the observed associations.

## Data Availability

The original contributions presented in the study are included in the article/**Supplementary Material**. Further inquiries can be directed to the corresponding author.
