# Supplementary material for: Calm in the midst of the storm: inferring gene expression stability in flowering plants
Source: Front Plant Sci. 2026 Aug 17;17:1846139. doi: 10.3389/fpls.2026.1846139 (PMC13527180; doi:10.3389/fpls.2026.1846139)
Supplement: Supplementary file 1 [file Image1.pdf]

Supplementary Figures

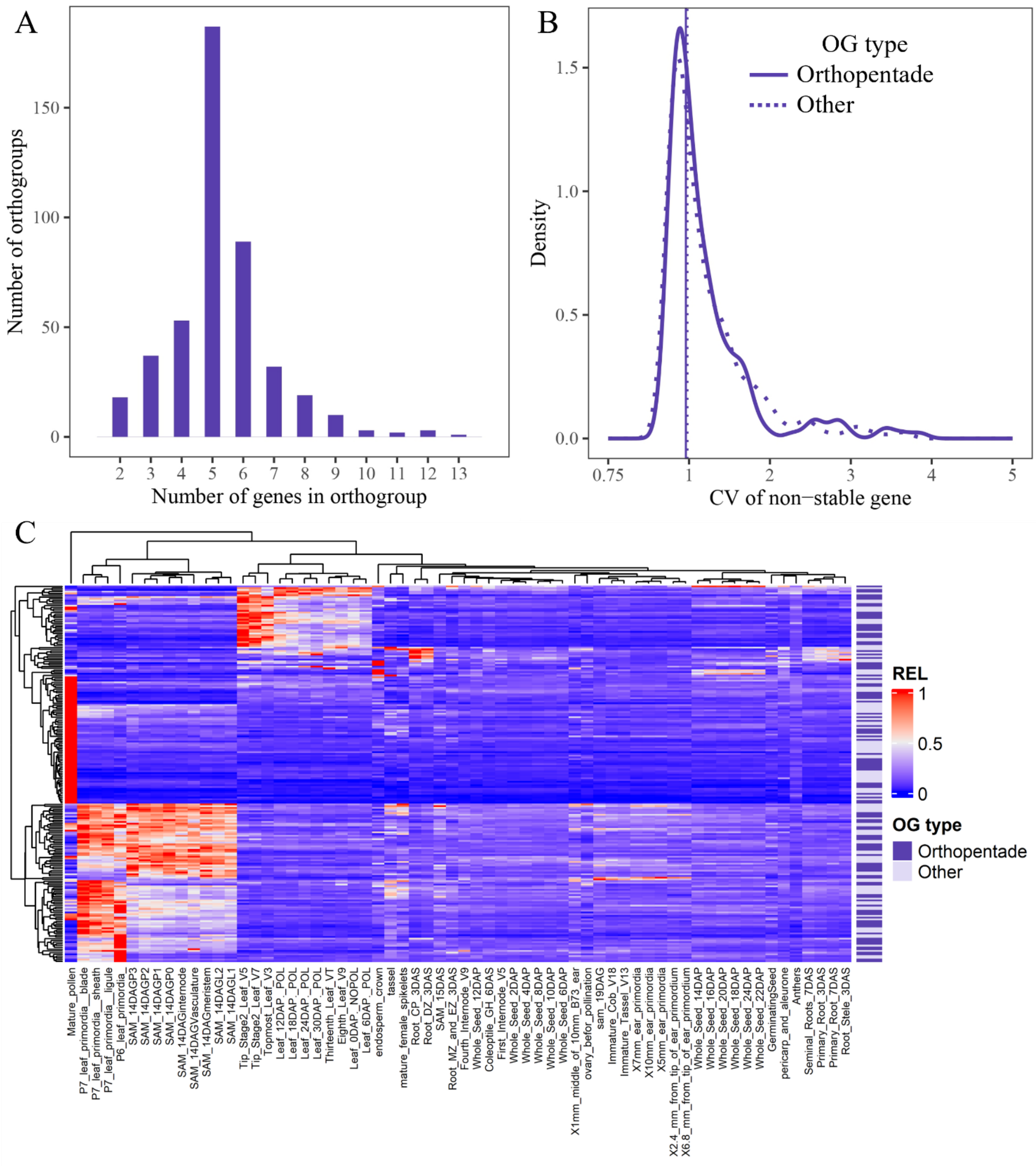

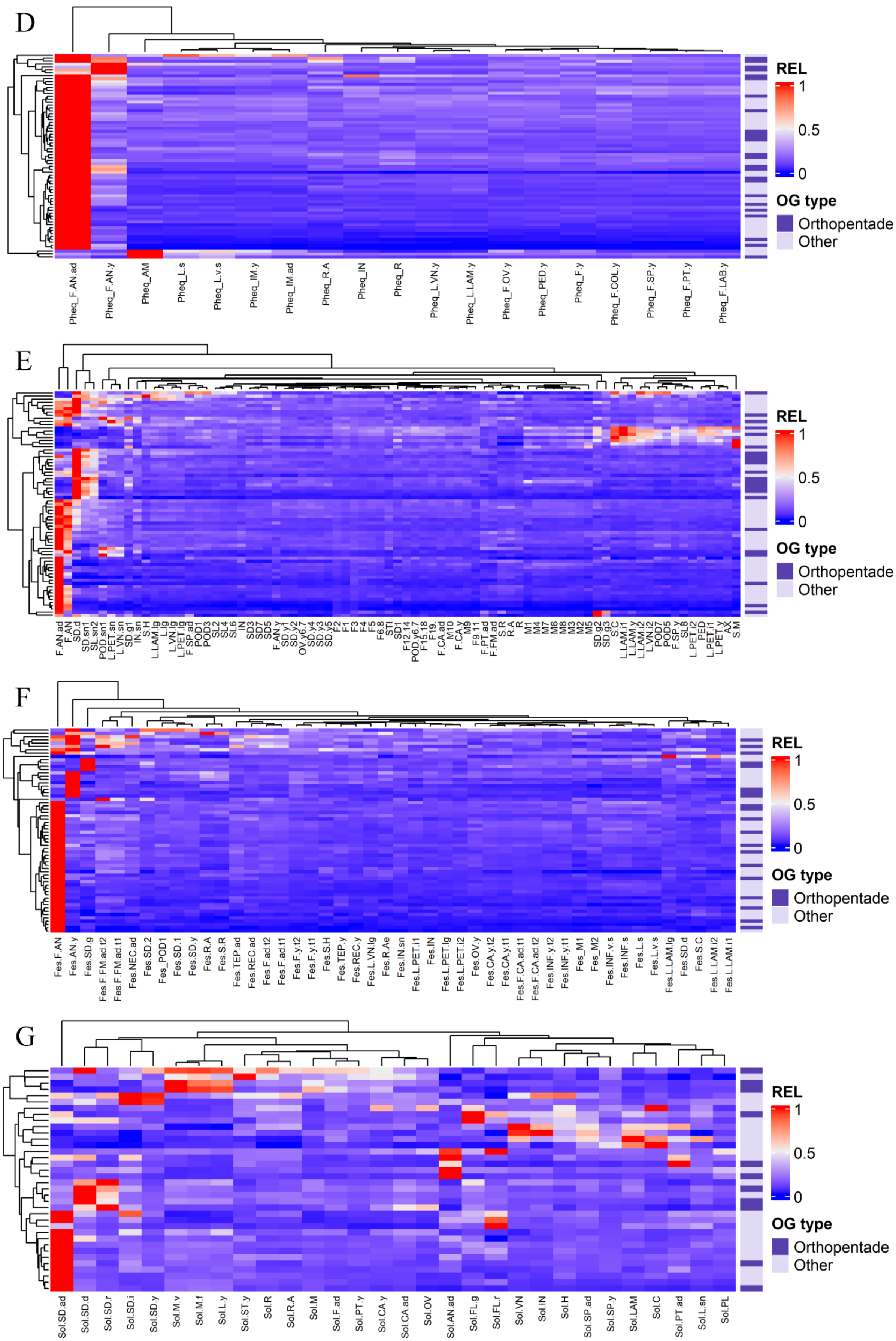
D
REL
1
0.5
0
OG type
Orthopentade
Other
Pheq_F.AN.ad
Pheq_F.AN.y
Pheq_AM
Pheq_L.s
Pheq_L.v.s
Pheq_IM.y
Pheq_IM.ad
Pheq_R.A
Pheq_IN
Pheq_R
Pheq_L.VN.y
Pheq_L.LAM.y
Pheq_F.OV.y
Pheq_PED.y
Pheq_F.y
Pheq_F.COL.y
Pheq_F.SP.y
Pheq_F.PT.y
Pheq_F.LAB.y
E
REL
1
0.5
0
OG type
Orthopentade
Other
F
REL
1
0.5
0
OG type
Orthopentade
Other
Fes.F.AN
Fes.AN.y
Fes.SD.g
Fes.F.FM.ad.t2
Fes.F.FM.ad.t1
Fes.NEC.ad
Fes.SD.2
Fes_POD1
Fes.SD.1
Fes.SD.y
Fes.R.A
Fes.S.R
Fes.TEP.ad
Fes.REC.ad
Fes.F.ad.t2
Fes.F.ad.t1
Fes.F.y.t2
Fes.F.y.t1
Fes.S.H
Fes.TEP.y
Fes.REC.y
Fes.L.VN.lg
Fes.R.Ae
Fes.IN.sn
Fes.L.PET.i1
Fes.IN
Fes.L.PET.lg
Fes.L.PET.i2
Fes.OV.y
Fes.CA.y.t2
Fes.CA.y.t1
Fes.F.CA.ad.t1
Fes.F.CA.ad.t2
Fes.INF.y.t2
Fes.INF.y.t1
Fes_M1
Fes_M2
Fes.INF.v.s
Fes.INF.s
Fes.L.s
Fes.L.v.s
Fes.L.LAM.lg
Fes.SD.d
Fes.S.C
Fes.L.LAM.i2
Fes.L.LAM.i1
G
REL
1
0.5
0
OG type
Orthopentade
Other
Sol.SD.ad
Sol.SD.d
Sol.SD.r
Sol.SD.i
Sol.SD.y
Sol.M.v
Sol.M.f
Sol.L.y
Sol.ST.y
Sol.R
Sol.R.A
Sol.M
Sol.F.ad
Sol.PT.y
Sol.CA.y
Sol.CA.ad
Sol.OV
Sol.AN.ad
Sol.FL.g
Sol.FL.r
Sol.VN
Sol.IN
Sol.H
Sol.SP.ad
Sol.SP.y
Sol.LAM
Sol.C
Sol.PT.ad
Sol.L.sn
Sol.PL

Supplementary Figure 1. Orthogroups with all but one stable genes. **(A)** A distribution of size of orthogroups with all but one stable genes considering only five "core" species. **(B)** A CV distribution of unstable genes belonging to orthogroups with all but one stable genes. Orthopentades and other orthogroups are pictured separately. Vertical lines represent the first quartile of distributions: solid line for orthopentades and dotted line for other orthogroups. **(C-G)** Heatmaps of expression levels of unstable genes from orthogroups with all but one stable genes of **(C)** *Z.mays*, **(D)** *P. equestris*, **(E)** *A. thaliana*, **(F)** *F. esculentum*, **(G)** *S. lycopersicum*. REL stands for relative expression level: for each gene expression level was measured in normalized on library size readcounts, then readcounts were averaged over biological replicates of each sample and was divided by the maximal expression level of a gene. Orthopentades and other orthogroups are marked at the right-sided annotation named "OG type".

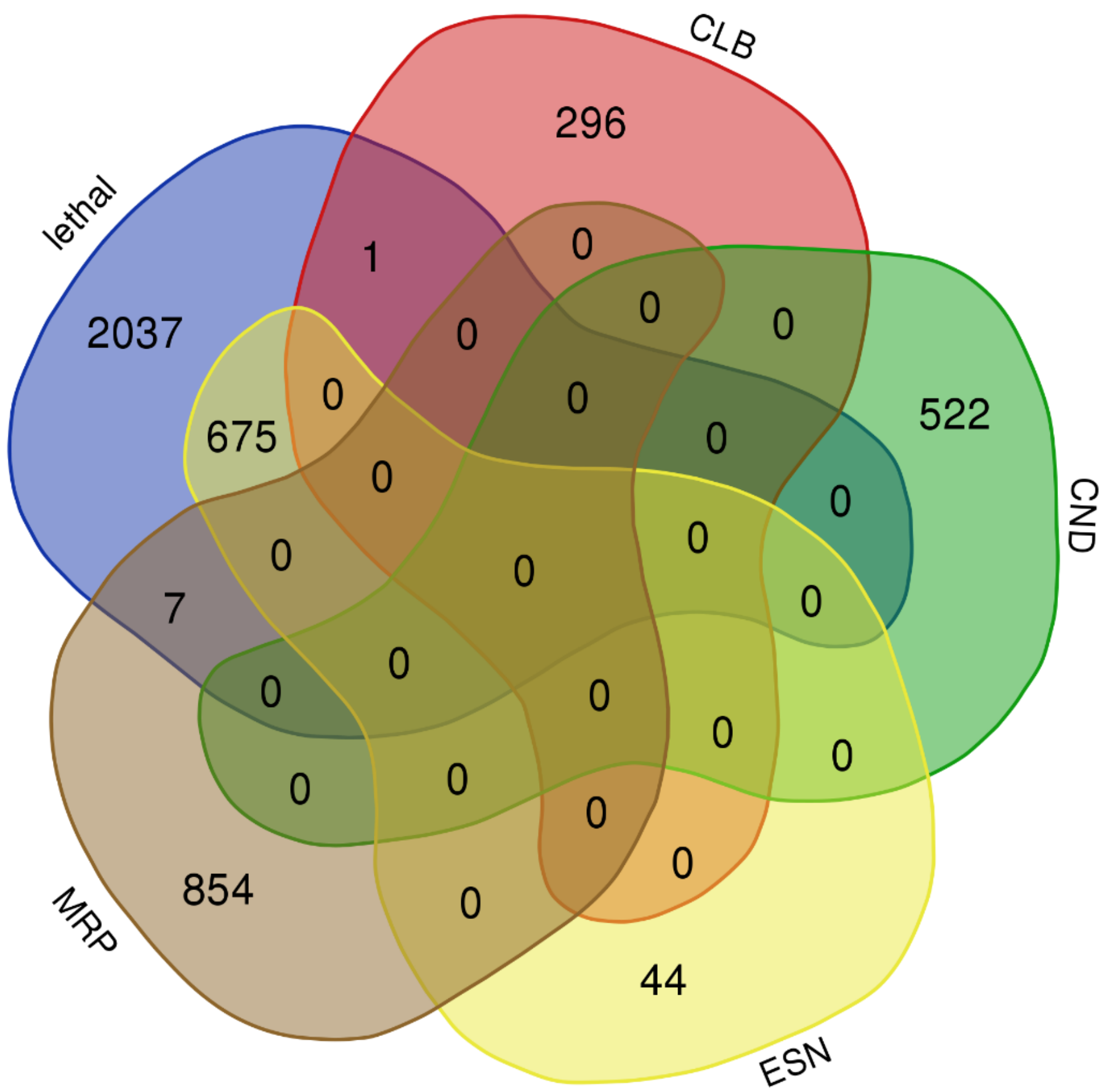

Supplementary Figure 2. Venn diagram of *A. thaliana* genes identified as lethal when loss-of-function mutation occurs (Lloyd et al., 2015) and *A. thaliana* genes described as "essential" (ESN, genes loss-of-function mutations in which lead to lethality), "morphological" (MRP, genes loss-of-function mutations in which lead to morphological defects in a plant), "cellular biochemical defects" (CLB) and "conditional defects" (CND) in (Lloyd and Meinke, 2012).

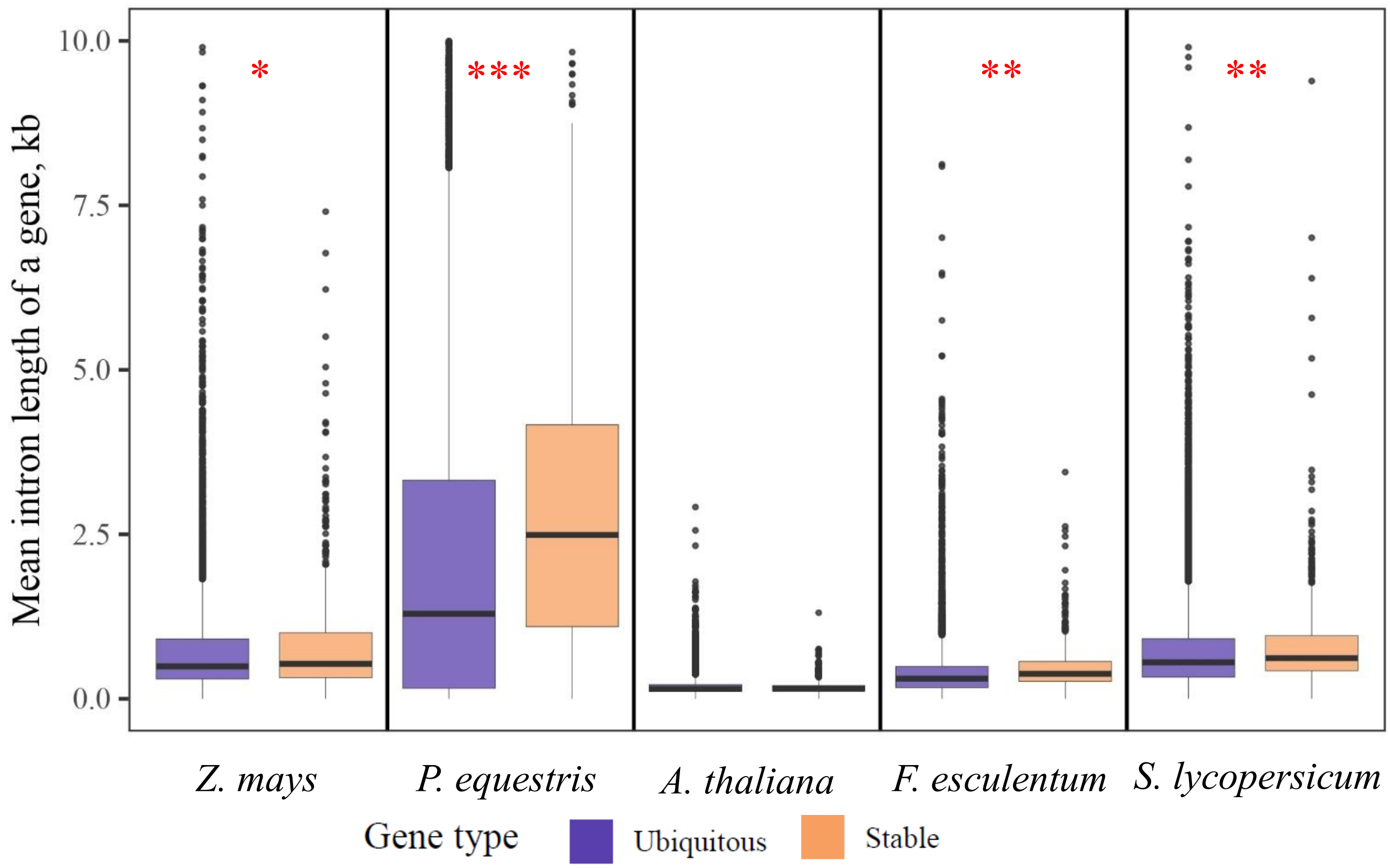

Supplementary Figure 3. Mean intron length of stable and ubiquitous genes for each species. Asterisks indicate statistical significance of difference between distributions of expressed and stable genes using Mann-Whitney test with *** for FDR-corrected p-value lower than 1.0e-10; ** - 1.0e-10 < FDR < 1.0e-5; * 1.0e-5 < FDR < 0.05.

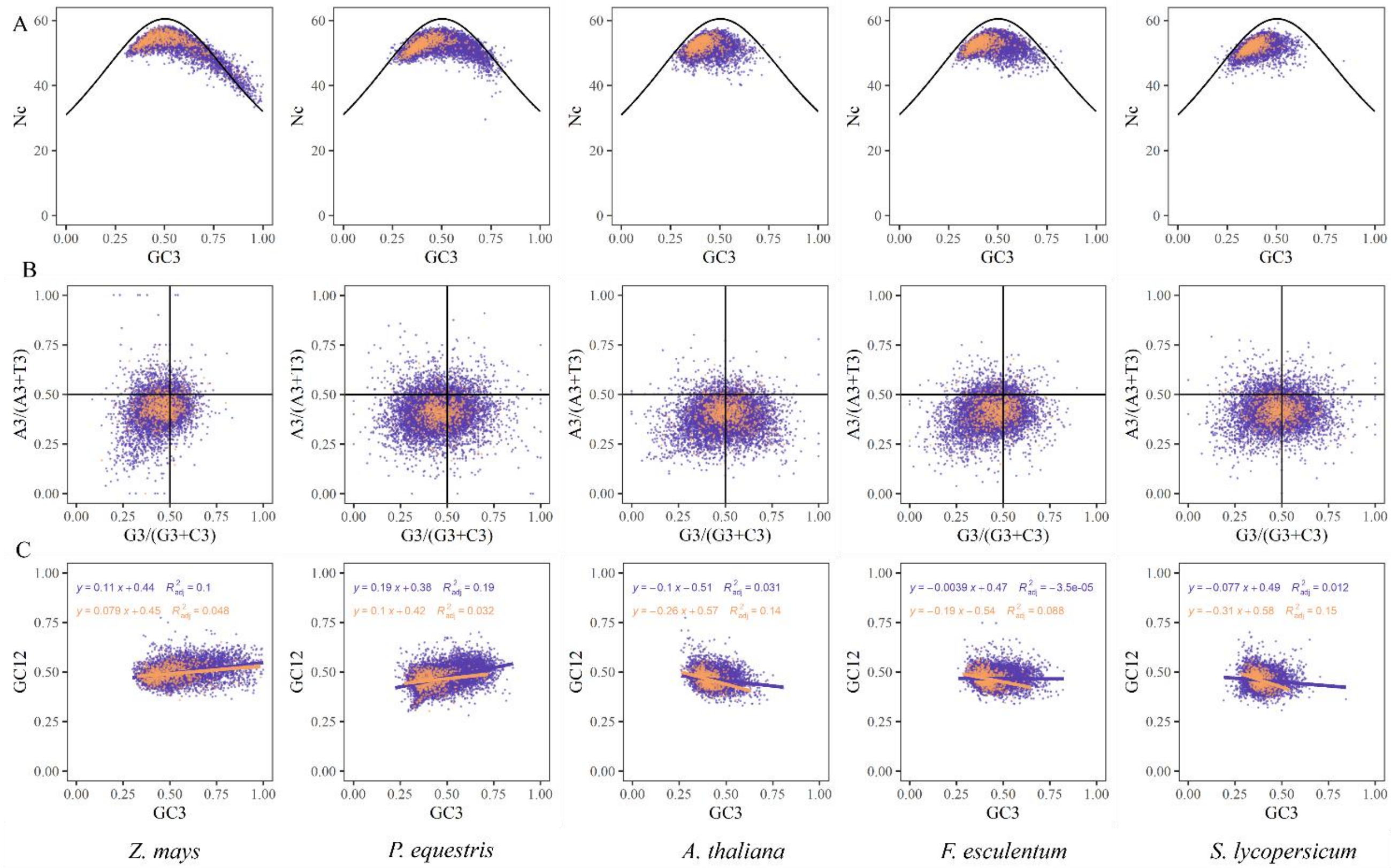

Supplementary Figure 4. The characteristics of codon usage of expressed and stable genes. **(A)** Nc-GC3 plot for ubiquitous and stable genes of five species. Solid line represents expected effective number of codons (Nc) values for a given GC3 (GC content at degenerative position of codon) in the absence of natural selection (Wright, 1990). **(B)** PR2 bias plot for ubiquitous and stable genes of five species. Codon usage bias resulting from mutational process only should lead to the uniform presence of GC or AT for four-fold degenerate amino acids, which is a consequence of type 2 parity rule (PR2 (Sueoka, 1995)). **(C)** Neutrality plot for expressed and stable genes of five species. Neutrality plot represents the relationship between GC12 (GC content in non-degenerative positions) and GC3 (GC content in degenerative position) content. If GC12 and GC3 are correlated and the slope of the regression line is close to one, the main source of codon bias is the mutational process (Sueoka, 1988), in other case natural selection is dominant. Lines represent linear regression for expressed and stable genes separately. Linear regression formulas and R^2 are given for each regression line.

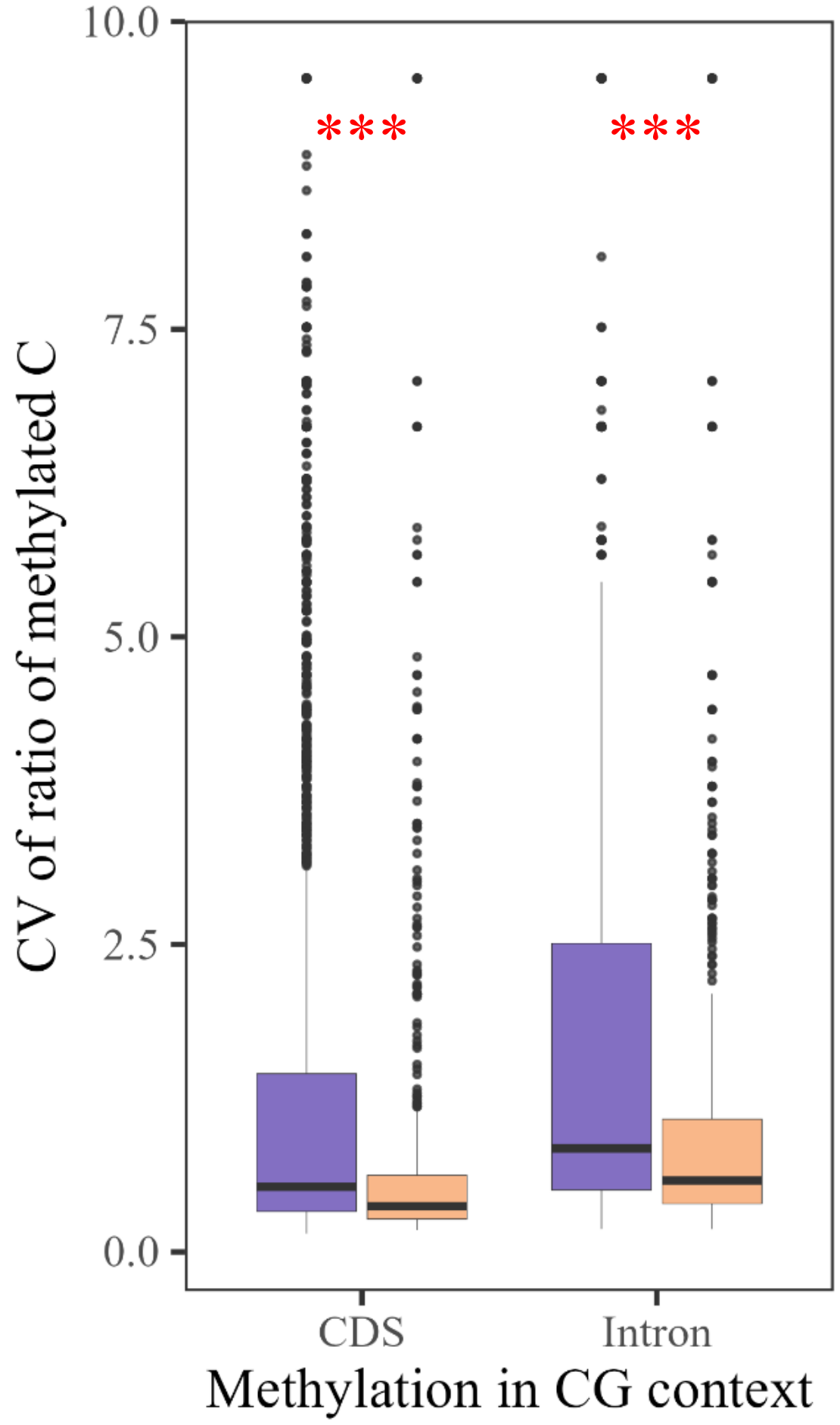

Supplementary Figure 5. Gene cytosine methylation. Coefficient of variance (CV) across 91 samples of ratio of methylated cytosines to all cytosines in CG context in CDS and introns of ubiquitous and stable *A. thaliana* genes. Asterisks indicate statistical significance of difference between distributions of expressed and stable genes using Mann-Whitney test with *** for FDR-corrected p-value lower than 1.0e-10; ** - 1.0e-10 < FDR < 1.0e-5; * 1.0e-5 < FDR < 0.05.

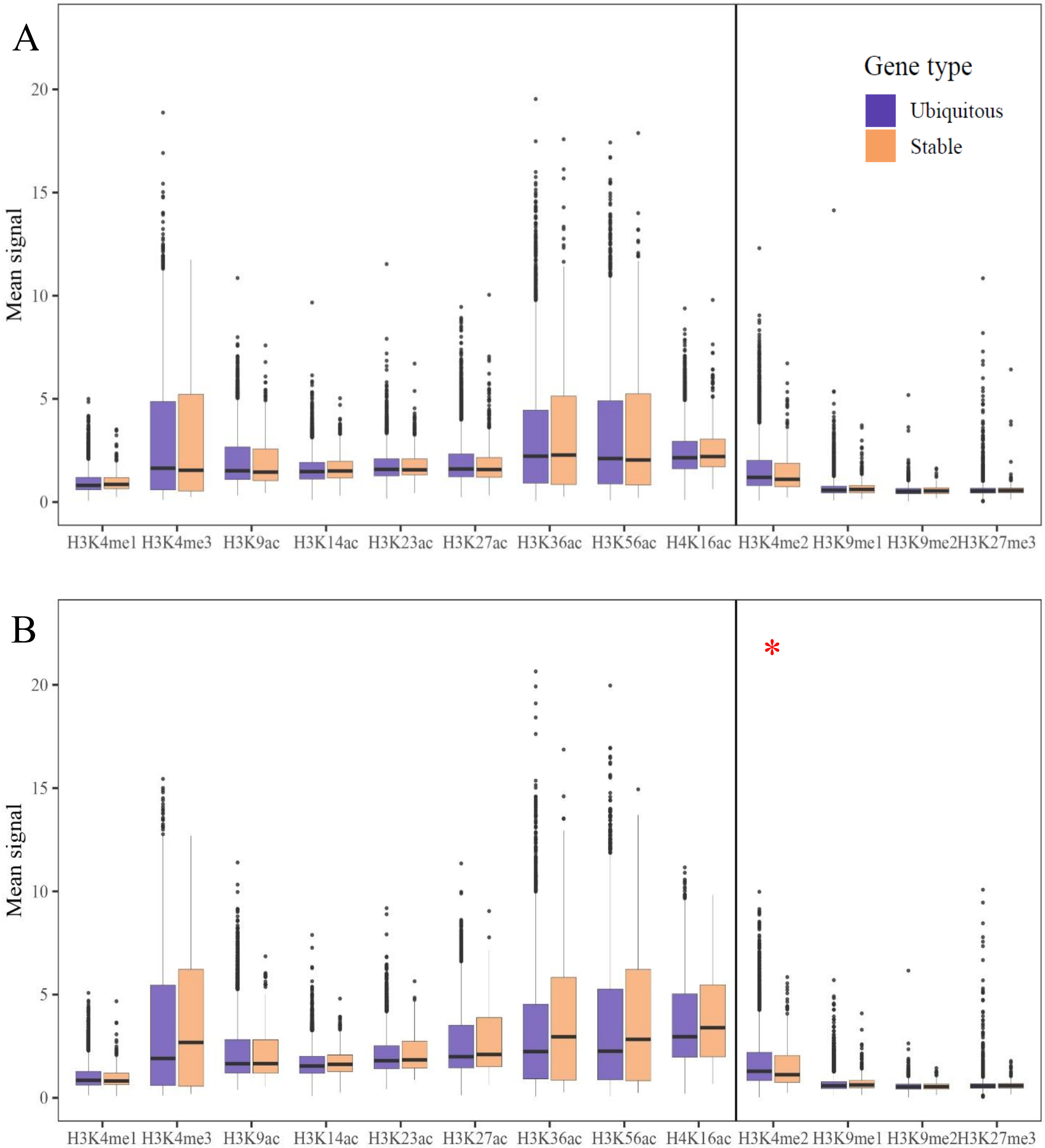

Supplementary Figure 6. Histone marks in non-coding regions. **(A)** The distribution of histone modifications in 5'-UTR of ubiquitous and stable *A. thaliana* genes. **(B)** The distribution of histone modifications in 3'-UTR of ubiquitous and stable *A. thaliana* genes. Vertical solid line separates active histone marks (left part) from repressive ones (right part). Asterisks indicate statistical significance of difference between distributions of expressed and stable genes using Mann-Whitney test with *** for FDR-corrected p-value lower than 1.0e-10; ** - 1.0e-10 < FDR < 1.0e-5; * 1.0e-5 < FDR < 0.05.

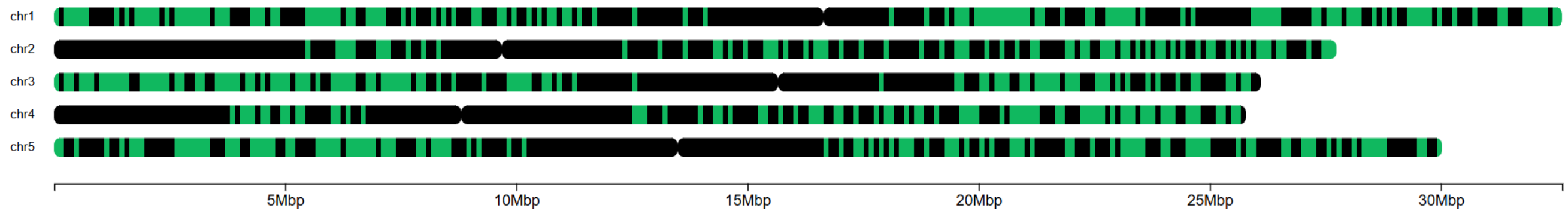

Supplementary Figure 7. The distribution of SEGs across *A. thaliana* chromosomes. Gene location is colored green.
