# Supplementary material for: Calm in the midst of the storm: inferring gene expression stability in flowering plants
Source: Front Plant Sci. 2026 Aug 17;17:1846139. doi: 10.3389/fpls.2026.1846139 (PMC13527180; doi:10.3389/fpls.2026.1846139)
Supplement: Supplementary file 2 [file DataSheet1.docx]

Supplementary File 1

# 1 Angiosperm genome assemblies used in the analysis

We selected 109 assemblies of Angiosperm genomes from Phytozome 14 (Goodstein et al., 2012) (see the full list of assemblies in Table 1 of Supplementary File 1). We combined 109 species with “core” for this study five species (*Arabidopsis*, buckwheat, *Phalaenopsis*, tomato, and maize) and used OrthoFinder (Emms and Kelly, 2019) to identify gene orthogroups.

**Table 1 of Supplementary File 1.** The list of genome assemblies of Angiosperm plants used for orthologization.

| Species | Downloaded file |
| --- | --- |
| *Acorus americanus* | Aamericanus_586_v1.1.protein_primaryTranscriptOnly |
| *Aquilegia coerulea* | Acoerulea_322_v3.1.protein_primaryTranscriptOnly |
| *Ananas comosus* | Acomosus_321_v3.protein_primaryTranscriptOnly |
| *Arabidopsis halleri* | Ahalleri_264_v1.1.protein_primaryTranscriptOnly |
| *Amaranthus hypochondriacus* | Ahypochondriacus_459_v2.1.protein_primaryTranscriptOnly |
| *Arachis hypogaea* | AhypogaeaLine8_886_v1.3.protein_primaryTranscriptOnly |
| *Alyssum linifolium* | Alinifolium_472_v1.1.protein_primaryTranscriptOnly |
| *Arabidopsis lyrata* | Alyrata_384_v2.1.protein_primaryTranscriptOnly |
| *Anacardium occidentale* | Aoccidentale_449_v0.9.protein_primaryTranscriptOnly |
| *Asparagus officinalis* | Aofficinalis_498_V1.1.protein_primaryTranscriptOnly |
| *Amborella trichopoda* | Atrichopoda_291_v1.0.protein_primaryTranscriptOnly |
| *Brachypodium distachyon* | Bdistachyon_556_v3.2.protein_primaryTranscriptOnly |
| *Brachypodium hybridum* | BhybridumBhyb26_693_v2.1.protein_primaryTranscriptOnly |
| *Brassica oleracea capitata* | Boleraceacapitata_446_v1.0.protein_primaryTranscriptOnly |
| *Betula platyphylla* | Bplatyphylla_679_v1.1.protein_primaryTranscriptOnly |
| *Brassica rapa* | BrapaFPsc_277_v1.3.protein_primaryTranscriptOnly |
| *Boechera stricta* | Bstricta_278_v1.2.protein_primaryTranscriptOnly |
| *Beta vulgaris* | Bvulgarisssp_vulgaris_782_EL10.2_2.protein_primaryTranscriptOnly |
| *Caulanthus amplexicaulis* | Camplexicaulis_470_v1.1.protein_primaryTranscriptOnly |
| *Cicer arietinum* | Carietinum_492_v1.0.protein_primaryTranscriptOnly |
| *Corymbia citriodora* | Ccitriodora_507_v2.1.protein_primaryTranscriptOnly |
| *Citrus clementina* | Cclementina_182_protein_primaryTranscriptOnly |
| *Castanea dentata* | Cdentata_673_v1.1.protein_primaryTranscriptOnly |
| *Capsella grandiflora* | Cgrandiflora_266_v1.1.protein_primaryTranscriptOnly |
| *Crambe hispanica* | Chispanica_488_v1.1.protein_primaryTranscriptOnly |
| *Carya illinoinensis* | Cillinoinensis_573_v1.1.protein_primaryTranscriptOnly |
| *Cinnamomum kanehirae* | Ckanehirae_531_v3.protein_primaryTranscriptOnly |
| *Chasmanthium laxum* | Claxum_690_v1.1.protein_primaryTranscriptOnly |
| *Cakile maritima* | Cmaritima_481_v1.1.protein_primaryTranscriptOnly |
| *Carica papaya* | Cpapaya_113_ASGPBv0.4.protein_primaryTranscriptOnly |
| *Chenopodium quinoa* | Cquinoa_392_v1.0.protein_primaryTranscriptOnly |
| *Capsella rubella* | Crubella_474_v1.1.protein_primaryTranscriptOnly |
| *Cucumis sativus* | Csativus_122_protein_primaryTranscriptOnly |
| *Citrus sinensis* | Csinensis_154_protein_primaryTranscriptOnly |
| *Cleome violacea* | Cviolacea_585_v2.1.protein_primaryTranscriptOnly |
| *Dioscorea alata* | Dalata_550_v2.1.protein_primaryTranscriptOnly |
| *Daucus carota* | Dcarotasubsp_sativus_861_v3.0.protein_primaryTranscriptOnly |
| *Descurainia sophioides* | Dsophioides_482_v1.1.protein_primaryTranscriptOnly |
| *Diptychocarpus strictus* | Dstrictus_582_v2.1.protein_primaryTranscriptOnly |
| *Eucalyptus grandis* | Egrandisvar_TAG0014HAP2_994_v4.2.protein_primaryTranscriptOnly |
| *Eutrema salsugineum* | Esalsugineum_173_v1.0.protein_primaryTranscriptOnly |
| *Euclidium syriacum* | Esyriacum_483_v1.1.protein_primaryTranscriptOnly |
| *Eruca vesicaria* | Evesicaria_487_v1.1.protein_primaryTranscriptOnly |
| *Fragaria vesca* | Fvesca_677_v4.0.a2.protein_primaryTranscriptOnly |
| *Gossypium hirsutum* | Ghirsutum_578_v3.1.protein_primaryTranscriptOnly |
| *Glycine max* | Gmax_880_Wm82.a6.v1.protein_primaryTranscriptOnly |
| *Gossypium raimondii* | Graimondii_221_protein_primaryTranscriptOnly |
| *Glycine soja* | Gsoja_509_v1.1.protein_primaryTranscriptOnly |
| *Helianthus annuus* | Hannuus_494_r1.2.protein_primaryTranscriptOnly |
| *Hydrangea quercifolia* | Hquercifolia_589_v1.1.protein_primaryTranscriptOnly |
| *Hordeum vulgare* | HvulgareMorex_702_V3.protein_primaryTranscriptOnly |
| *Iberis amara* | Iamara_485_v1.1.protein_primaryTranscriptOnly |
| *Isatis tinctoria* | Itinctoria_475_v1.1.protein_primaryTranscriptOnly |
| *Joinvillea ascendens* | Jascendens_587_v1.1.protein_primaryTranscriptOnly |
| *Kalanchoe fedtschenkoi* | Kfedtschenkoi_382_v1.1.protein_primaryTranscriptOnly |
| *Kalanchoe laxiflora* | Klaxiflora_309_v1.1.protein_primaryTranscriptOnly |
| *Lupinus albus* | Lalbus_567_v1.protein_primaryTranscriptOnly |
| *Lunaria annua* | Lannua_476_v1.1.protein_primaryTranscriptOnly |
| *Lotus japonicus* | LjaponicusGifu_1046_v1.3.protein_primaryTranscriptOnly |
| *Lindenbergia philippensis* | Lphilippensis_689_v1.1.protein_primaryTranscriptOnly |
| *Lactuca sativa* | Lsativa_467_v5.protein_primaryTranscriptOnly |
| *Lepidium sativum* | Lsativum_478_v1.1.protein_primaryTranscriptOnly |
| *Linum usitatissimum* | Lusitatissimum_200_protein_primaryTranscriptOnly |
| *Musa acuminata* | Macuminata_304_v1.protein_primaryTranscriptOnly |
| *Malus domestica* | Mdomestica_491_v1.1.protein_primaryTranscriptOnly |
| *Manihot esculenta* | Mesculenta_671_v8.1.protein_primaryTranscriptOnly |
| *Mimulus guttatus* | MguttatusTOL_551_v5.0.protein_primaryTranscriptOnly |
| *Malcolmia maritima* | Mmaritima_477_v1.1.protein_primaryTranscriptOnly |
| *Myagrum perfoliatum* | Mperfoliatum_583_v2.1.protein_primaryTranscriptOnly |
| *Miscanthus sinensis* | Msinensis_497_v7.1.protein_primaryTranscriptOnly |
| *Medicago truncatula* | Mtruncatula_285_Mt4.0v1.protein_primaryTranscriptOnly |
| *Nymphaea colorata* | Ncolorata_566_v1.2.protein_primaryTranscriptOnly |
| *Olea europaea* | Oeuropaea_451_v1.0.protein_primaryTranscriptOnly |
| *Oryza sativa* | Osativa_323_v7.0.protein_primaryTranscriptOnly |
| *Oropetium thomaeum* | Othomaeum_386_v1.0.protein_primaryTranscriptOnly |
| *Phaseolus acutifolius* | PacutifoliusWLD_581_v2.0.protein_primaryTranscriptOnly |
| *Portulaca amilis* | Pamilis_692_v1.0.protein_primaryTranscriptOnly |
| *Populus deltoides* | PdeltoidesWV94_445_v2.1.protein_primaryTranscriptOnly |
| *Panicum hallii* | Phallii_590_v3.2.protein_primaryTranscriptOnly |
| *Pharus latifolius* | Platifolius_680_v1.1.protein_primaryTranscriptOnly |
| *Phaseolus lunatus* | Plunatus_563_V1.protein_primaryTranscriptOnly |
| *Prunus persica* | Ppersica_298_v2.1.protein_primaryTranscriptOnly |
| *Populus trichocarpa* | Ptrichocarpa_533_v4.1.protein_primaryTranscriptOnly |
| *Poncirus trifoliata* | Ptrifoliata_565_v1.3.1.protein_primaryTranscriptOnly |
| *Paspalum vaginatum* | Pvaginatum_672_v3.1.protein_primaryTranscriptOnly |
| *Panicum virgatum* | Pvirgatum_516_v5.1.protein_primaryTranscriptOnly |
| *Phaseolus vulgaris* | Pvulgaris_442_v2.1.protein_primaryTranscriptOnly |
| *Quercus rubra* | Qrubra_687_v2.1.protein_primaryTranscriptOnly |
| *Ricinus communis* | Rcommunis_119_v0.1.protein_primaryTranscriptOnly |
| *Rorippa islandica* | Rislandica_473_v1.1.protein_primaryTranscriptOnly |
| *Sinapis alba* | Salba_584_v3.1.protein_primaryTranscriptOnly |
| *Sinapis alba* | Sbicolor_730_v5.1.protein_primaryTranscriptOnly |
| *Setaria italica* | Sitalica_312_v2.2.protein_primaryTranscriptOnly |
| *Spinacia oleracea* | Soleracea_575_Spov3.protein_primaryTranscriptOnly |
| *Schrenkiella parvula* | Sparvula_574_v2.2.protein_primaryTranscriptOnly |
| *Stanleya pinnata* | Spinnata_486_v1.1.protein_primaryTranscriptOnly |
| *Spirodela polyrhiza* | Spolyrhiza_290_v2.protein_primaryTranscriptOnly |
| *Salix purpurea* | Spurpurea_519_v5.1.protein_primaryTranscriptOnly |
| *Solanum tuberosum* | Stuberosum_686_v6.1.protein_primaryTranscriptOnly |
| *Setaria viridis* | Sviridis_726_v4.1.protein_primaryTranscriptOnly |
| *Thlaspi arvense v1.1* | Tarvense_479_v1.1.protein_primaryTranscriptOnly |
| *Theobroma cacao* | Tcacao_523_v2.1.protein_primaryTranscriptOnly |
| *Thinopyrum intermedium* | Tintermedium_770_v3.1.protein_primaryTranscriptOnly |
| *Trifolium pratense* | Tpratense_385_v2.protein_primaryTranscriptOnly |
| *Urochloa fusca* | Ufusca_669_v1.1.protein_primaryTranscriptOnly |
| *Vaccinium darrowii* | Vdarrowii_700_v1.2.protein_primaryTranscriptOnly |
| *Vigna unguiculata* | Vunguiculata_540_v1.2.protein_primaryTranscriptOnly |
| *Vitis vinifera* | Vvinifera_457_v2.1.protein_primaryTranscriptOnly |
| *Zostera marina* | Zmarina_668_v3.1.protein_primaryTranscriptOnly |

We obtained 71 989 orthogroups. The number of genes in the orthogroups varied between two and 10 647 (4 077 244 genes in total; single-gene orthogroups were excluded at the previous stage), while the number of species in the orthogroups was 1-114. We excluded single-species orthogroups, after that 45 900 orthogroups remained. The sizes of these orthogroups are presented at Figure 1 of Supplementary File 1.

**Figure 1 of Supplementary File 1** The distribution of orthogroup size (single-genes and single-species orthogroups removed).

# 2 References

Emms, D. M., and Kelly, S. (2019). OrthoFinder: phylogenetic orthology inference for comparative genomics. *Genome Biol* 20, 238. doi: 10.1186/s13059-019-1832-y

Goodstein, D. M., Shu, S., Howson, R., Neupane, R., Hayes, R. D., Fazo, J., et al. (2012). Phytozome: a comparative platform for green plant genomics. *Nucleic Acids Research* 40, D1178–D1186. doi: 10.1093/nar/gkr944
