# Supplementary material for: Calm in the midst of the storm: inferring gene expression stability in flowering plants
Source: Front Plant Sci. 2026 Aug 17;17:1846139. doi: 10.3389/fpls.2026.1846139 (PMC13527180; doi:10.3389/fpls.2026.1846139)
Supplement: Supplementary file 3 [file DataSheet2.docx]

Supplementary File 2

# 1 Mapping statistics of transcriptome maps used in the analysis

The detailed transcriptome maps are described in the following papers: *Zea mays* (Stelpflug et al., 2016), *Phalaenopsis equestris* (Klepikova et al., 2021), *Arabidopsis thaliana* (Klepikova et al., 2016), *Fagopyrum esculentum* (Penin et al., 2021), and *Solanum lycopersicum* (Penin et al., 2019) The full list of transcriptome map samples used in the study see Supplementary Table 1. Mapping statistics for all samples are given in the Table 1 of Supplementary File 1.

**Table 1 of Supplementary File 1.** Number of uniquely mapped on genes reads in transcriptome maps samples used in the study.

| *A. thaliana* | | *F. esculentum* | | *Ph. equestris* | | *S. lycopersicum* | | *Z. mays* | |
| --- | --- | --- | --- | --- | --- | --- | --- | --- | --- |
| Sample | Mapped reads | Sample | Mapped reads | Sample | Mapped reads | Sample | Mapped reads | Sample | Mapped reads |
| SRR3581857 | 24824919 | SRR7758458 | 15428575 | SRR13734103 | 22104584 | Sol.ST.y_2 | 13681900 | SRR1573504 | 17828388 |
| SRR3581704 | 22285037 | SRR7758474 | 18853238 | SRR13734085 | 37952325 | Sol.SD.y_1 | 19227311 | SRR1573515 | 17334095 |
| SRR3581878 | 24128617 | SRR7758444 | 14272748 | SRR13734106 | 19742849 | Sol.SP.ad_1 | 20543838 | SRR531908 | 10191059 |
| SRR3581388 | 20867076 | SRR7758411 | 15337525 | SRR13734111 | 22124032 | Sol.R_1 | 14042711 | SRR531904 | 10673475 |
| SRR3581894 | 35254859 | SRR7758396 | 30488909 | SRR13734086 | 35044716 | Sol.F.ad_2 | 18207100 | SRR1619668 | 20637082 |
| SRR3581848 | 24935153 | SRR7758462 | 15821099 | SRR13734117 | 16582399 | Sol.FL.g_1 | 20663255 | SRR531895 | 18900986 |
| SRR3581854 | 24343346 | SRR7758463 | 17426893 | SRR13734101 | 22415529 | Sol.OV_2 | 15072312 | SRR531869 | 26798869 |
| SRR3581891 | 20601311 | SRR7758412 | 15237418 | SRR13734093 | 23073585 | Sol.SD.ad_1 | 20347944 | SRR531944 | 22970606 |
| SRR3581881 | 25618908 | SRR7758470 | 14165246 | SRR13734107 | 20593202 | Sol.IN_2 | 16562244 | SRR940293 | 17609472 |
| SRR3581899 | 26311463 | SRR7758397 | 25940762 | L.VN.y_1 | 15771030 | Sol.PT.y_2 | 20122430 | SRR940284 | 17080957 |
| SRR3581686 | 21232124 | SRR7758420 | 23625876 | SRR13734109 | 18937433 | Sol.AN.ad_2 | 14142928 | SRR1573507 | 12358436 |
| SRR3581876 | 22429478 | SRR7758436 | 17132350 | L.LAM.y_2 | 15755067 | Sol.L.sn_2 | 18713660 | SRR5310815 | 17384980 |
| SRR1688425 | 22419764 | SRR7758408 | 16005613 | SRR13734096 | 23017402 | Sol.SP.ad_2 | 21981542 | SRR999034 | 18645118 |
| SRR3581684 | 21180659 | SRR7758398 | 14848042 | SRR13734108 | 26545319 | Sol.H_1 | 14125302 | SRR940239 | 13435606 |
| SRR1661476 | 31990876 | SRR7758469 | 14972457 | SRR13734095 | 33730257 | Sol.M_2 | 19548731 | SRR531220 | 19749339 |
| SRR3581849 | 21077269 | SRR7758392 | 21950778 | SRR13734089 | 18077132 | Sol.M.v_1 | 21686818 | SRR5456940 | 54141594 |
| SRR3581860 | 23494462 | SRR7758413 | 22628264 | SRR13734110 | 25765108 | Sol.FL.g_2 | 18599052 | SRR531870 | 17532162 |
| SRR3581877 | 27547654 | SRR7758389 | 22793122 | SRR13734094 | 24618106 | Sol.R_2 | 13686043 | SRR5310818 | 17664735 |
| SRR3581846 | 22780368 | SRR7758414 | 15593944 | SRR13734116 | 22993638 | Sol.SD.ad_2 | 16375633 | SRR957468 | 18291195 |
| SRR3581732 | 28050131 | SRR7758465 | 16034759 | SRR13734113 | 20555065 | Sol.SD.d_2 | 17333746 | SRR5310809 | 18132986 |
| SRR3581892 | 24726220 | SRR7758394 | 23907382 | SRR13734097 | 26559755 | Sol.R.A_1 | 17001731 | SRR1619637 | 17431914 |
| SRR1688426 | 21151804 | SRR7758448 | 13651911 | SRR13734088 | 26898321 | Sol.VN_2 | 13932038 | SRR1619667 | 19943682 |
| SRR3581717 | 22078361 | SRR7758391 | 13979370 | SRR13734100 | 24534894 | Sol.LAM_1 | 12966575 | SRR957445 | 18327166 |
| SRR3581865 | 24623640 | SRR7758454 | 12898374 | SRR13734114 | 25732758 | Sol.LAM_2 | 20384109 | SRR504480 | 17512387 |
| SRR3581706 | 22485133 | SRR7758390 | 21882998 | SRR13734115 | 23091121 | Sol.R.A_2 | 23313037 | SRR531880 | 15693505 |
| SRR3581863 | 24505311 | SRR7758421 | 13871390 | SRR13734120 | 24081254 | Sol.AN.ad_1 | 25634443 | SRR1619633 | 10752846 |
| SRR3581719 | 21346547 | SRR7758432 | 15105995 | SRR13734105 | 24484385 | Sol.L.y_2 | 19800721 | SRR957474 | 10525813 |
| SRR3581855 | 23997274 | SRR7758395 | 22376298 | SRR13734098 | 25339149 | Sol.PT.ad_1 | 14426488 | SRR3098750 | 23633559 |
| SRR1688328 | 21005962 | SRR7758459 | 15842933 | SRR13734121 | 21412025 | Sol.OV_1 | 13667031 | SRR531900 | 13863723 |
| SRR3581708 | 21805926 | SRR7758445 | 12314258 | SRR13734104 | 22545260 | Sol.H_2 | 14902607 | SRR5310826 | 16553770 |
| SRR3581834 | 23630085 | SRR7758416 | 14832541 | L.LAM.y_1 | 24495577 | Sol.PL_1 | 14346763 | SRR531876 | 13218518 |
| SRR3581870 | 20736058 | SRR7758455 | 14115649 | SRR13734090 | 25576761 | Sol.PT.ad_2 | 14974762 | SRR957420 | 17775913 |
| SRR3581896 | 27070818 | SRR7758429 | 16104963 | SRR13734102 | 18827561 | Sol.CA.y_2 | 14823850 | SRR5310823 | 18966798 |
| SRR3581895 | 23472746 | SRR7758417 | 13669412 | SRR13734092 | 16959929 | Sol.SP.y_1 | 11056751 | SRR1620940 | 15177043 |
| SRR3581839 | 22859362 | SRR7758452 | 15614353 | SRR13734112 | 19205361 | Sol.SD.r_1 | 15994754 | SRR957449 | 17624692 |
| SRR3581862 | 22137674 | SRR7758471 | 16138336 | SRR13734091 | 24391929 | Sol.SD.i_1 | 20569109 | SRR1633397 | 16156160 |
| SRR3581735 | 27513406 | SRR7758425 | 12295493 | L.VN.y_2 | 18435148 | Sol.CA.y_1 | 14693113 | SRR957423 | 13943577 |
| SRR3581499 | 22366820 | SRR7758447 | 11590293 | SRR13734099 | 24519907 | Sol.PL_2 | 21531133 | SRR5674387 | 27284687 |
| SRR3581869 | 28998909 | SRR7758406 | 15009181 |  |  | Sol.VN_1 | 17253200 | SRR5674385 | 12782608 |
| SRR3581850 | 25902493 | SRR7758419 | 12999795 |  |  | Sol.M.f_2 | 14730420 | SRR1620939 | 10531553 |
| SRR3581859 | 22477932 | SRR7758402 | 12551124 |  |  | Sol.M.v_2 | 15399751 | SRR5674384 | 19458480 |
| SRR3581713 | 20557469 | SRR7758457 | 17616553 |  |  | Sol.M_1 | 17345275 | SRR5674380 | 28063270 |
| SRR3581688 | 22075560 | SRR7758473 | 20853246 |  |  | Sol.ST.y_1 | 15593813 | SRR957422 | 10230226 |
| SRR3581733 | 22438842 | SRR7758424 | 15659009 |  |  | Sol.IN_1 | 17275855 | SRR531879 | 14533964 |
| SRR3581727 | 23820469 | SRR7758393 | 16813129 |  |  | Sol.L.y_1 | 19010410 | SRR1620952 | 11080324 |
| SRR3581716 | 24345507 | SRR7758418 | 11835727 |  |  | Sol.M.f_1 | 17629124 | SRR5674388 | 17269769 |
| SRR3581712 | 24619005 | SRR7758437 | 13847976 |  |  | Sol.PT.y_1 | 21170470 | SRR404188 | 10350137 |
| SRR1659921 | 21439337 | SRR7758428 | 17987517 |  |  | Sol.C_2 | 14734468 | SRR404139 | 18567172 |
| SRR3581694 | 21873061 | SRR7758404 | 33215576 |  |  | Sol.SD.d_1 | 10755419 | SRR504481 | 14944610 |
| SRR3581336 | 21014077 | SRR7758451 | 15653365 |  |  | Sol.C_1 | 23815563 | SRR5456959 | 15975563 |
| SRR3581838 | 26008419 | SRR7758440 | 13105695 |  |  | Sol.SP.y_2 | 22644932 | SRR1573509 | 16334387 |
| SRR1661477 | 34466236 | SRR7758461 | 12923632 |  |  | Sol.L.sn_1 | 18593284 | SRR531910 | 12859127 |
| SRR3581893 | 36304708 | SRR7758409 | 13774315 |  |  | Sol.SD.y_2 | 16332877 | SRR940283 | 10741590 |
| SRR3581872 | 21469034 | SRR7758439 | 21929898 |  |  | Sol.CA.ad_1 | 22517713 | SRR531219 | 17328998 |
| SRR3581842 | 20228553 | SRR7758438 | 11880808 |  |  | Sol.CA.ad_2 | 16098509 | SRR940259 | 23519894 |
| SRR3581346 | 23838629 | SRR7758468 | 15889534 |  |  | Sol.SD.r_2 | 17728014 | SRR531909 | 15153915 |
| SRR3581851 | 24482445 | SRR7758403 | 13349981 |  |  | Sol.SD.i_2 | 20686313 | SRR1573505 | 17900550 |
| SRR3581885 | 22254967 | SRR7758449 | 11604289 |  |  | Sol.F.ad_1 | 16099510 | SRR5310825 | 16831438 |
| SRR3581721 | 22888040 | SRR7758477 | 16173331 |  |  | Sol.FL.r_2 | 15058248 | SRR404140 | 17727809 |
| SRR3581858 | 24877480 | SRR7758460 | 21654670 |  |  | Sol.FL.r_1 | 19089578 | SRR1619661 | 19088572 |
| SRR3581682 | 23048869 | SRR7758441 | 21659871 |  |  |  |  | SRR1573511 | 16788215 |
| SRR3581835 | 22059649 | SRR7758467 | 12829853 |  |  |  |  | SRR957418 | 12324117 |
| SRR3581678 | 20827100 | SRR7758433 | 14072575 |  |  |  |  | SRR1573508 | 15374900 |
| SRR3581740 | 20460457 | SRR7758435 | 18390060 |  |  |  |  | SRR957446 | 16281988 |
| SRR3581887 | 25315162 | SRR7758400 | 15045096 |  |  |  |  | SRR531903 | 13301317 |
| SRR3581699 | 24539651 | SRR7758478 | 13245924 |  |  |  |  | SRR5310824 | 17333473 |
| SRR3581734 | 22226226 | SRR7758450 | 13602221 |  |  |  |  | SRR531898 | 19041340 |
| SRR3581886 | 24998585 | SRR7758456 | 16706830 |  |  |  |  | SRR3098752 | 19166057 |
| SRR3581738 | 22044062 | SRR7758405 | 19473261 |  |  |  |  | SRR531905 | 15672223 |
| SRR3581736 | 21751150 | SRR7758443 | 14227932 |  |  |  |  | SRR940290 | 13384188 |
| SRR1661478 | 25529794 | SRR7758415 | 16165634 |  |  |  |  | SRR531224 | 10753918 |
| SRR1661483 | 21174243 | SRR7758466 | 12686918 |  |  |  |  | SRR531922 | 17405787 |
| SRR3581868 | 23153563 | SRR7758442 | 16246630 |  |  |  |  | SRR5310813 | 11058086 |
| SRR3581347 | 21900149 | SRR7758426 | 20537931 |  |  |  |  | SRR531935 | 16725335 |
| SRR3581852 | 21817432 | SRR7758475 | 17267967 |  |  |  |  | SRR957450 | 19574440 |
| SRR3581700 | 24689618 | SRR7758422 | 13597976 |  |  |  |  | SRR5456961 | 11669116 |
| SRR3581897 | 25734646 | SRR7758407 | 13999986 |  |  |  |  | SRR1619664 | 19608182 |
| SRR3581879 | 21262930 | SRR7758464 | 16240790 |  |  |  |  | SRR531899 | 15533998 |
| SRR3581898 | 23057111 | SRR7758472 | 15238240 |  |  |  |  | SRR404181 | 15335661 |
| SRR3581692 | 21233727 | SRR7758479 | 15107540 |  |  |  |  | SRR1620938 | 12377231 |
| SRR3581875 | 19924111 | SRR7758423 | 14917019 |  |  |  |  | SRR5674381 | 16017745 |
| SRR3581720 | 21884838 | SRR7758434 | 15193798 |  |  |  |  | SRR1573514 | 15337796 |
| SRR3581696 | 20727854 | SRR7758446 | 21027208 |  |  |  |  | SRR5456939 | 33518921 |
| SRR3581890 | 23458774 | SRR7758430 | 13846449 |  |  |  |  | SRR1620954 | 10442627 |
| SRR3581695 | 23638438 | SRR7758453 | 14843018 |  |  |  |  | SRR940274 | 15850338 |
| SRR3581679 | 22480306 | SRR7758410 | 15066936 |  |  |  |  | SRR531896 | 18615484 |
| SRR1688327 | 21158495 | SRR7758388 | 13366265 |  |  |  |  | SRR404194 | 17510113 |
| SRR3581847 | 23569717 | SRR7758431 | 12845699 |  |  |  |  | SRR531886 | 11191801 |
| SRR3581873 | 22460900 | SRR7758427 | 12651178 |  |  |  |  | SRR531924 | 16982985 |
| SRR3581864 | 20637135 | SRR7758399 | 24670589 |  |  |  |  | SRR5674382 | 17561238 |
| SRR3581689 | 21041285 | SRR7758401 | 18990031 |  |  |  |  | SRR940260 | 14788954 |
| SRR1661484 | 20345511 | SRR7758476 | 14609522 |  |  |  |  | SRR957448 | 17316079 |
| SRR1688427 | 20781583 |  |  |  |  |  |  | SRR531934 | 19362697 |
| SRR3581356 | 21909068 |  |  |  |  |  |  | SRR531902 | 48228671 |
| SRR1661473 | 22220237 |  |  |  |  |  |  | SRR531942 | 25053608 |
| SRR3581737 | 21815887 |  |  |  |  |  |  | SRR999035 | 16962848 |
| SRR3581345 | 21852585 |  |  |  |  |  |  | SRR531226 | 13682869 |
| SRR3581853 | 29796229 |  |  |  |  |  |  | SRR531933 | 14759607 |
| SRR3581866 | 22488053 |  |  |  |  |  |  | SRR940286 | 12230128 |
| SRR1661474 | 23275742 |  |  |  |  |  |  | SRR531912 | 11835631 |
| SRR3581856 | 22276992 |  |  |  |  |  |  | SRR3098754 | 16546427 |
| SRR3581676 | 22203836 |  |  |  |  |  |  | SRR5310814 | 17823094 |
| SRR3581841 | 22167334 |  |  |  |  |  |  | SRR1620969 | 19788299 |
| SRR3581731 | 23840310 |  |  |  |  |  |  | SRR1626426 | 17845288 |
| SRR1688326 | 22388293 |  |  |  |  |  |  | SRR531888 | 17600670 |
| SRR3581709 | 23727804 |  |  |  |  |  |  | SRR5674386 | 19308557 |
| SRR3581685 | 21174757 |  |  |  |  |  |  | SRR5456960 | 15522765 |
| SRR1659922 | 35650510 |  |  |  |  |  |  | SRR1620920 | 18893317 |
| SRR3581883 | 22168698 |  |  |  |  |  |  | SRR5310816 | 18106549 |
| SRR3581702 | 22966991 |  |  |  |  |  |  | SRR1620966 | 40216159 |
| SRR3581383 | 25662324 |  |  |  |  |  |  | SRR957467 | 18784070 |
| SRR3581861 | 23444653 |  |  |  |  |  |  | SRR404132 | 13744309 |
| SRR3581639 | 23409376 |  |  |  |  |  |  | SRR5310808 | 18179646 |
| SRR1660398 | 30606132 |  |  |  |  |  |  | SRR531878 | 20993466 |
| SRR1660397 | 22278766 |  |  |  |  |  |  | SRR940237 | 13642715 |
| SRR3581672 | 23535934 |  |  |  |  |  |  | SRR940280 | 13211134 |
| SRR3581681 | 22958948 |  |  |  |  |  |  | SRR1619636 | 41382995 |
| SRR3581693 | 22639451 |  |  |  |  |  |  | SRR531877 | 11614983 |
| SRR3581683 | 22985076 |  |  |  |  |  |  | SRR5310812 | 15409535 |
| SRR3581726 | 20625261 |  |  |  |  |  |  | SRR5310822 | 16606808 |
| SRR3581698 | 22009816 |  |  |  |  |  |  | SRR1620917 | 12412943 |
| SRR3581680 | 22400341 |  |  |  |  |  |  | SRR1573510 | 24631640 |
| SRR3581874 | 26953940 |  |  |  |  |  |  | SRR531945 | 17290993 |
| SRR3581352 | 22562256 |  |  |  |  |  |  | SRR5674383 | 21597786 |
| SRR3581687 | 26574726 |  |  |  |  |  |  | SRR5310817 | 17138619 |
| SRR3581836 | 21621397 |  |  |  |  |  |  | SRR531887 | 17759501 |
| SRR1688325 | 20136188 |  |  |  |  |  |  | SRR5310810 | 18025432 |
| SRR3581711 | 20574799 |  |  |  |  |  |  | SRR1620973 | 31196410 |
| SRR3581724 | 23378163 |  |  |  |  |  |  | SRR1620948 | 16101958 |
| SRR3581837 | 24520732 |  |  |  |  |  |  | SRR1619671 | 17167286 |
| SRR3581728 | 26790930 |  |  |  |  |  |  | SRR531225 | 12100634 |
| SRR3581705 | 21346704 |  |  |  |  |  |  | SRR940291 | 19601454 |
| SRR3581845 | 22765290 |  |  |  |  |  |  | SRR404179 | 18854501 |
| SRR3581690 | 22639716 |  |  |  |  |  |  | SRR1573512 | 22814945 |
| SRR3581714 | 20302923 |  |  |  |  |  |  | SRR957473 | 17712272 |
| SRR3581882 | 20711986 |  |  |  |  |  |  | SRR531906 | 14122900 |
| SRR3581715 | 25360163 |  |  |  |  |  |  | SRR1620956 | 10671776 |
| SRR3581888 | 21228428 |  |  |  |  |  |  | SRR5310821 | 17842828 |
| SRR3581701 | 21338681 |  |  |  |  |  |  | SRR1620955 | 11037767 |
| SRR3581591 | 29896757 |  |  |  |  |  |  | SRR5310811 | 19184533 |
| SRR3581840 | 20858130 |  |  |  |  |  |  | SRR531911 | 14875595 |
| SRR3581707 | 22081348 |  |  |  |  |  |  | SRR1619660 | 19261859 |
| SRR3581871 | 21138602 |  |  |  |  |  |  | SRR531201 | 22903307 |
| SRR3581703 | 21468673 |  |  |  |  |  |  | SRR1619634 | 14364506 |
| SRR3581697 | 21826379 |  |  |  |  |  |  | SRR957466 | 19407644 |
| SRR1661475 | 39287569 |  |  |  |  |  |  | SRR1619674 | 16958882 |
| SRR3581710 | 22014301 |  |  |  |  |  |  | SRR940238 | 14650614 |
| SRR3581884 | 21938505 |  |  |  |  |  |  | SRR531218 | 14195711 |
| SRR3581844 | 25603760 |  |  |  |  |  |  | SRR531881 | 21152538 |
| SRR3581831 | 30075969 |  |  |  |  |  |  | SRR1573506 | 16066445 |
| SRR3581691 | 21843195 |  |  |  |  |  |  | SRR957421 | 18607074 |
| SRR3581833 | 22074666 |  |  |  |  |  |  | SRR531203 | 22519795 |
| SRR3581889 | 23540061 |  |  |  |  |  |  | SRR1620959 | 18961028 |
| SRR3581880 | 20828696 |  |  |  |  |  |  | SRR531871 | 18223552 |
| SRR3581867 | 20612292 |  |  |  |  |  |  | SRR957419 | 12293336 |
| SRR1688429 | 20535203 |  |  |  |  |  |  | SRR1620968 | 11293861 |
| SRR3581730 | 25958023 |  |  |  |  |  |  | SRR5310820 | 16726671 |
| SRR3581843 | 21684808 |  |  |  |  |  |  | SRR1620949 | 15544871 |
|  |  |  |  |  |  |  |  | SRR1573513 | 10264981 |
|  |  |  |  |  |  |  |  | SRR531901 | 14739690 |
|  |  |  |  |  |  |  |  | SRR531925 | 14504028 |
|  |  |  |  |  |  |  |  | SRR531907 | 17827588 |
|  |  |  |  |  |  |  |  | SRR531875 | 13046748 |
|  |  |  |  |  |  |  |  | SRR1620947 | 15782688 |
|  |  |  |  |  |  |  |  | SRR957447 | 20252581 |
|  |  |  |  |  |  |  |  | SRR404196 | 17294631 |
|  |  |  |  |  |  |  |  | SRR531897 | 18490484 |
|  |  |  |  |  |  |  |  | SRR1620960 | 12130141 |

# 2 Sample correlation in transcriptome maps

We analyzed the quality of transcriptome maps in terms of biological replicates similarity using hierarchical clustering of samples. In all studied species biological replicates showed high correlation (Figure 1 of Supplementary File 1).

A

B

C

D

E

**Figure 1 of Supplementary File 2** Hierarchical clustering trees of transcriptome map samples. (A) *A. thaliana*, (B) *F. esculentum*, (C) *Ph. equestris*, (D) *S. lycopersicum*, (E) *Z. mays*.

# 3 Batch-effect in transcriptome maps

The presence of batch-effects was analyzed with PCA. For all transcriptome maps PCA reflects biological variability of plant samples (Figure 2 of Supplementary File 2)

**Figure 2 of Supplementary File 2** PCA of transcriptome map samples.

# 4 References

Klepikova, A. V., Kasianov, A. S., Ezhova, M. A., Penin, A. A., and Logacheva, M. D. (2021). Transcriptome atlas of *Phalaenopsis equestris*. *PeerJ* 9, e12600. doi: 10.7717/peerj.12600

Klepikova, A. V., Kasianov, A. S., Gerasimov, E. S., Logacheva, M. D., and Penin, A. A. (2016). A High Resolution Map of the *Arabidopsis thaliana* Developmental Transcriptome Based on RNA-seq Profiling. *The Plant Journal*. doi: 10.1111/tpj.13312

Penin, A. A., Kasianov, A. S., Klepikova, A. V., Kirov, I. V., Gerasimov, E. S., Fesenko, A. N., et al. (2021). High-Resolution Transcriptome Atlas and Improved Genome Assembly of Common Buckwheat, Fagopyrum esculentum. *Front. Plant Sci.* 12, 612382. doi: 10.3389/fpls.2021.612382

Penin, A. A., Klepikova, A. V., Kasianov, A. S., Gerasimov, E. S., and Logacheva, M. D. (2019). Comparative Analysis of Developmental Transcriptome Maps of Arabidopsis thaliana and Solanum lycopersicum. *Genes* 10, 50. doi: 10.3390/genes10010050

Stelpflug, S. C., Sekhon, R. S., Vaillancourt, B., Hirsch, C. N., Buell, C. R., de Leon, N., et al. (2016). An Expanded Maize Gene Expression Atlas based on RNA Sequencing and its Use to Explore Root Development. *The Plant Genome* 9, 0. doi: 10.3835/plantgenome2015.04.0025
