# Supplementary material for: Calm in the midst of the storm: inferring gene expression stability in flowering plants
Source: Front Plant Sci. 2026 Aug 17;17:1846139. doi: 10.3389/fpls.2026.1846139 (PMC13527180; doi:10.3389/fpls.2026.1846139)
Supplement: Supplementary file 4 [file DataSheet3.docx]

Supplementary File 3

# 1 The analysis of *Cucumis sativa* RNA-seq data

In order to explore gene expression stability of *Cucumis sativa* we selected 104 RNA-seq experiments belonging to eight BioProjects of the NCBI SRA database. The samples are listed in Table 1 of Supplementary File S2. RNA-seq reads were mapped on *C. sativa* reference genome (Phytozome 14) and number of reads mapped at each gene were counted. Samples with total number of reads mapped on genes lower than 10M and higher than 40M were excluded from the analysis. After that 84 samples remained. We normalized gene expression at library size as in “DESeq” (Anders and Huber, 2010), selected genes that were expressed in all samples (with a threshold 16 or more normalized reads per gene) and calculated CV for each gene.

**Table 1 of Supplementary File 2.** The list of *C. sativa* RNA-seq experiments. For each SRR the corresponding BioProject is listed as well as whether the SRR is used in the subsequent analysis.

| BioProject | Run | Was the sample excluded from the analysis? |
| --- | --- | --- |
| PRJNA672817 | SRR12919474 | No |
| PRJNA672817 | SRR12919475 | No |
| PRJNA672817 | SRR12919476 | No |
| PRJNA672817 | SRR12919477 | No |
| PRJNA672817 | SRR12919478 | No |
| PRJNA672817 | SRR12919479 | No |
| PRJNA672817 | SRR12919480 | No |
| PRJNA672817 | SRR12919481 | No |
| PRJNA672817 | SRR12919482 | No |
| PRJNA672817 | SRR12919483 | No |
| PRJNA672817 | SRR12919484 | No |
| PRJNA672817 | SRR12919485 | No |
| PRJNA672817 | SRR12919486 | No |
| PRJNA672817 | SRR12919487 | No |
| PRJNA672817 | SRR12919488 | No |
| PRJNA672817 | SRR12919489 | No |
| PRJNA672817 | SRR12919490 | No |
| PRJNA672817 | SRR12919491 | No |
| PRJNA718963 | SRR14126825 | No |
| PRJNA718963 | SRR14126826 | No |
| PRJNA718963 | SRR14126827 | No |
| PRJNA718963 | SRR14126828 | No |
| PRJNA718963 | SRR14126829 | No |
| PRJNA718963 | SRR14126830 | No |
| PRJNA718963 | SRR14126831 | No |
| PRJNA718963 | SRR14126832 | No |
| PRJNA718963 | SRR14126833 | No |
| PRJNA718963 | SRR14126834 | No |
| PRJNA718963 | SRR14126835 | No |
| PRJNA718963 | SRR14126836 | No |
| PRJNA765376 | SRR16074103 | Yes |
| PRJNA765376 | SRR16074104 | Yes |
| PRJNA765376 | SRR16074105 | Yes |
| PRJNA765376 | SRR16074106 | Yes |
| PRJNA765376 | SRR16074107 | Yes |
| PRJNA765376 | SRR16074108 | Yes |
| PRJNA765376 | SRR16074109 | Yes |
| PRJNA765376 | SRR16074110 | Yes |
| PRJNA817708 | SRR18404684 | No |
| PRJNA817708 | SRR18404685 | No |
| PRJNA817708 | SRR18404686 | No |
| PRJNA817708 | SRR18404687 | No |
| PRJNA817708 | SRR18404688 | No |
| PRJNA817708 | SRR18404689 | No |
| PRJNA817708 | SRR18404690 | No |
| PRJNA817708 | SRR18404691 | No |
| PRJNA817708 | SRR18404692 | No |
| PRJNA817708 | SRR18404693 | No |
| PRJNA817708 | SRR18404694 | No |
| PRJNA817708 | SRR18404695 | No |
| PRJNA381300 | SRR5405122 | Yes |
| PRJNA381300 | SRR5405123 | Yes |
| PRJNA381300 | SRR5405124 | Yes |
| PRJNA381300 | SRR5405125 | Yes |
| PRJNA381300 | SRR5405126 | Yes |
| PRJNA381300 | SRR5405127 | Yes |
| PRJNA381300 | SRR5405128 | Yes |
| PRJNA381300 | SRR5405129 | Yes |
| PRJNA381300 | SRR6320532 | Yes |
| PRJNA381300 | SRR6320533 | Yes |
| PRJNA381300 | SRR6320534 | Yes |
| PRJNA381300 | SRR6320535 | Yes |
| PRJNA431715 | SRR6691636 | No |
| PRJNA431715 | SRR6691637 | No |
| PRJNA431715 | SRR6691638 | No |
| PRJNA431715 | SRR6691639 | No |
| PRJNA431715 | SRR6691640 | No |
| PRJNA431715 | SRR6691641 | No |
| PRJNA431715 | SRR6691642 | No |
| PRJNA431715 | SRR6691643 | No |
| PRJNA431715 | SRR6691644 | No |
| PRJNA431715 | SRR6691645 | No |
| PRJNA431715 | SRR6691646 | No |
| PRJNA431715 | SRR6691647 | No |
| PRJNA484083 | SRR7637113 | No |
| PRJNA484083 | SRR7637114 | No |
| PRJNA484083 | SRR7637115 | No |
| PRJNA484083 | SRR7637116 | No |
| PRJNA484083 | SRR7637117 | No |
| PRJNA484083 | SRR7637118 | No |
| PRJNA484083 | SRR7637119 | No |
| PRJNA484083 | SRR7637120 | No |
| PRJNA484083 | SRR7637121 | No |
| PRJNA484083 | SRR7637122 | No |
| PRJNA484083 | SRR7637123 | No |
| PRJNA484083 | SRR7637124 | No |
| PRJNA525253 | SRR8703827 | No |
| PRJNA525253 | SRR8703828 | No |
| PRJNA525253 | SRR8703829 | No |
| PRJNA525253 | SRR8703830 | No |
| PRJNA525253 | SRR8703831 | No |
| PRJNA525253 | SRR8703832 | No |
| PRJNA525253 | SRR8703833 | No |
| PRJNA525253 | SRR8703834 | No |
| PRJNA525253 | SRR8703835 | No |
| PRJNA525253 | SRR8703836 | No |
| PRJNA525253 | SRR8703837 | No |
| PRJNA525253 | SRR8703838 | No |
| PRJNA525253 | SRR8703839 | No |
| PRJNA525253 | SRR8703840 | No |
| PRJNA525253 | SRR8703841 | No |
| PRJNA525253 | SRR8703842 | No |
| PRJNA525253 | SRR8703843 | No |
| PRJNA525253 | SRR8703844 | No |

# 2 References

Anders, S., and Huber, W. (2010). Differential expression analysis for sequence count data. *Genome Biol.* 11, R106. doi: 10.1186/gb-2010-11-10-r106
